# Promote or prevent? A regulatory focus perspective on managerial risk taking

**DOI:** 10.1371/journal.pone.0352905

**Published:** 2026-07-31

**Authors:** Amadeusz Jacek Miązek, Justyna Światowiec-Szczepańska

**Affiliations:** 1 Department of Management Accounting, Poznań University of Economics and Business, Poznań, Poland; 2 Department of Strategic Analysis, Krakow University of Economics, Kraków, Poland; PLOS: Public Library of Science, UNITED KINGDOM OF GREAT BRITAIN AND NORTHERN IRELAND

## Abstract

This study examines the heterogeneity of CEOs’ risk attitudes and how fixed versus variable compensation components shape their strategic risk-taking, as part of corporate governance policy. Building on psychological Regulatory Focus Theory (RFT) and established management frameworks, we developed a conceptual model with six hypotheses. We applied a validated Polish Linguistic Inquiry and Word Count (LIWC) dictionary to measure CEOs’ promotion and prevention focus through content analysis of their shareholder letters. Our dataset covers 82 companies listed on the Warsaw Stock Exchange (WSE) from 2011 to 2020, analyzed using longitudinal panel data. The results confirmed that a CEO’s regulatory focus is a significant motivational factor in strategic risk-taking. Promotion-focused CEOs tend to pursue bolder, riskier decisions, increasing the firm’s strategic risk, whereas prevention-focused CEOs are more cautious and inclined to mitigate risk, aligning with prior findings in the literature. Moreover, these relationships are moderated by compensation structure. Higher fixed salaries are associated with reduced strategic risk, reinforcing risk aversion, while larger annual bonuses are associated with greater risk-taking. In particular, fixed compensation strengthened the natural risk-avoidant tendencies of prevention-focused CEOs, suggesting that higher guaranteed income reinforces a cautious strategy. Unexpectedly, large annual bonuses did not temper the risk appetite of promotion-focused CEOs; instead, bonuses amplified their risk-taking, indicating that intrinsic motivation can outweigh extrinsic incentives for risk moderation. These findings underscore the need for tailoring executive compensation policies to individual CEOs’ risk preferences. Fixed salaries may temper excessive risk-taking in promotion-focused CEOs, while performance-based bonuses may motivate otherwise cautious, prevention-focused CEOs to undertake strategic risks. Such insights are valuable for refining corporate governance strategies in European public firms, especially in Poland, to better align CEO behavior with shareholder interests.

## Introduction

Strategic risk-taking is crucial for management practice and a key focus in strategic management research [1,2]. It involves leaders making proactive decisions about resource allocation that come with significant uncertainty and potential for substantial loss [3–6]. Executives must take these risks to enhance competitive positioning and company performance, though such decisions often introduce uncertainty [3,7].

Research consistently identifies the CEO as the most influential figure within an organization, exerting control over strategic risk, including revenue risks (e.g., [8–10]). The CEO’s authority gives them a unique advantage in guiding strategic decisions compared to other top managers [2,11,12]. This influence extends to the company’s ability to meet stakeholder needs [9]. A study by Reingold and Borrus [13] found that 64% of management professionals believe a company’s success or failure is largely determined by CEO decisions, a finding supported by research attributing up to 45% of a firm’s performance to the CEO [14].

Despite some variations in these findings, the consensus is that CEOs significantly shape the company’s risk profile and performance (e.g., [3]). Understanding what drives their risk-laden decisions is essential for gauging the level of risk expected by shareholders. Traditionally, early strategic management theories, like agency theory (AT) [15], focused on situational factors, particularly within the American corporate model. More recently, behavioral and psychological theories have expanded this view, including the Behavioral Agency Model (BAM) [16], Prospect Theory (PT) [17], and Regulatory Focus Theory (RFT) [18], which consider how decision-makers deviate from the status quo. RFT, in particular, suggests that individuals with a promotion or prevention focus may see the status quo differently, as either desirable or undesirable. Interest in individual determinants gained momentum with Hambrick and Mason’s [10] Upper Echelons Theory (UET), later updated by Hambrick [19], and the first applications of RFT in managerial risk-taking research emerged in 2015 [20].

UET posits that organizational outcomes reflect the cognitive and value-based characteristics of top executives [10]. Although this framework highlights observable attributes such as age, tenure, and educational background, it has been criticized for insufficiently addressing psychological traits that directly influence executive behavior. We extend UET by incorporating RFT, which captures chronic motivational orientation – promotion or prevention – that shapes how CEOs approach risk [18].

PT, on the other hand, provides a situational explanation of risk behavior based on whether outcomes are framed as gains or losses [17]. RFT complements this perspective by explaining stable individual differences in sensitivity to gain versus loss domains, independent of framing [21]. Promotion-focused CEOs are more attuned to gains and may exhibit higher risk tolerance, while prevention-focused CEOs prioritize security and loss avoidance. Our framework integrates these insights, suggesting that dispositional motivation (via RFT) and contextual framing (via PT) jointly shape strategic risk preferences. This layered approach enables a more granular understanding of how stable, dispositional traits (via RFT), observable background characteristics (via UET), and contextual frames (via PT) jointly influence risk-taking at the strategic apex. By nesting RFT within these frameworks, we not only enhance explanatory depth but also contribute to a growing effort to psychologize corporate governance.

While previous governance-oriented research has explored how different forms of CEO power – structural, expert, or prestige – affect strategic decision-making and firm outcomes [22], our approach shifts the analytical lens from formal power structures to internal motivational states. This contrast is important: power-based frameworks emphasize how CEOs influence organizational choices through position, control, or expertise, whereas our model focuses on how CEOs’ chronic regulatory focus drives their interpretation and enactment of incentive mechanisms. As such, we complement structural theories of governance with a psychological explanation of behavioral variability under identical formal conditions.

These theoretical developments intersect with practical concerns about how to design executive incentives that align with both firm strategy and individual traits. Practically, public companies use mixed executive compensation strategies that blend fixed and variable components. For example, in 2019, variable pay made up 19% of executive compensation at Warsaw Stock Exchange (WSE) companies, with increases following legislative changes and pandemic-driven adjustments [23]. Compensation ratios vary, being highest in the U.S. (CEO-to-employee pay ratio of 265x) and lower in, e.g., Poland (20.5x) [23,24]. This raises strategic questions about designing effective executive pay policies that consider CEOs’ diverse risk attitudes.

These insights reveal a significant research gap: how to integrate psychological theories of individual motivation into studies on situational factors like CEO compensation, particularly in European corporate contexts. These gaps inform our research agenda and guide three focal questions that structure the study: (1) Do regulatory foci affect strategic risk in European firms? (2) How do fixed and variable compensation components moderate this relationship? (3) Which elements of CEO pay exert the strongest influence on risk preferences?

The main hypothesis suggests regulatory focus is the most direct factor influencing CEOs’ impact on risk-taking, moderated by compensation elements like bonuses and fixed pay. From this, six hypotheses were developed. The first two propose a direct effect of CEOs’ promotion and prevention focus on strategic risk: positive for promotion, negative for prevention. The third and fourth hypotheses suggest annual bonuses weaken this link, while the fifth and sixth indicate that fixed pay strengthens it.

Prior studies of CEO risk-taking have largely relied on AT, BAM, and UET to explain variance in firm-level risk preferences [10,15,16]. These frameworks focus on incentive alignment, loss aversion, and managerial discretion. However, they typically underemphasize stable motivational traits that shape how executives perceive and respond to risk. RFT offers a dispositional lens that captures individual-level motivational orientation (promotion or prevention) that may drive risk behavior beyond situational framing [18,20].

While previous studies have linked compensation to CEO risk preferences [25,26], few have integrated such psychological orientations with corporate governance structures. This gap is particularly notable in European contexts. Our study addresses this by combining RFT with BAM and extending both UET and PT [17], offering a more comprehensive account of CEO decision-making under uncertainty. Unlike traditional governance theories that assume homogeneous executive responses to incentive structures, our framework introduces motivational asymmetry as a key explanatory mechanism. It thus advances behavioral governance by showing that the same incentive (e.g., a bonus) can be interpreted and enacted differently depending on the CEO’s chronic regulatory orientation.

Despite these theoretical advancements, prior empirical studies often suffer from limitations in integrating motivational and structural perspectives. Most large-sample analyses of CEO risk behavior rely on U.S.-based data and emphasize agency problems, risk incentives, or observable CEO characteristics (e.g., [25,27]). Even when RFT is considered, it is typically examined in isolation from corporate governance settings or treated as a marginal moderator [20,28]. In contrast, qualitative studies exploring psychological drivers (e.g., [29,30]) lack generalizability across institutional systems. Our study addresses this gap by developing and testing a moderated model in a corporate environment marked by hybrid governance logic [31,32]. By situating RFT within a multi-theoretical framework and linking it to specific incentive mechanisms (fixed and variable pay), we offer an empirically grounded, cross-contextual explanation of strategic risk-taking that advances the integration of psychological theory and governance research.

While this study is rooted in the Polish corporate environment, its theoretical framing and empirical approach draw relevance from broader European governance contexts. Poland’s governance model aligns with Central and Eastern European economies, but diverges from models in countries like Germany or Sweden, where stakeholder influence, co-determination, and stronger institutional investor activism are more pronounced [31–33]. Consequently, while the empirical focus remains on Poland, the study offers transferable insights for other EU member states navigating hybrid governance environments. In particular, our model may be relevant for economies undergoing a shift from concentrated ownership or state influence toward more shareholder-driven governance.

CEO compensation systems across Europe vary not only in magnitude but also in structure and philosophical underpinnings. While Poland relies on cash-based incentives (which stems, among other things, from the limited use and reporting of equity-based instruments in Poland prior to 2020) and retains elements of Anglo-Saxon pay-for-performance culture, countries like Germany adopt a co-determined, stakeholder-centric governance logic, which tempers risk-based incentives [34]. In contrast, Scandinavian systems often emphasize long-term value creation, low pay dispersion, and stewardship-based control, especially in family-controlled firms [35]. These models illustrate how institutional logics influence not only incentive design but also executive interpretation of strategic risk. These differences in compensation logic across European jurisdictions reinforce the importance of calibrating incentive structures not only to strategic objectives but also to institutional norms. Our framework may thus support boards and policymakers in designing more context-sensitive and psychologically attuned compensation strategies.

### Managerial risk

Risk is a fundamental element of managerial decision-making and a key focus in management research, including strategic management [36]. Despite progress in risk management theory and practice, defining risk remains challenging due to its ambiguity and normative nature [37]. In social sciences, risk is context-dependent and culturally influenced, requiring careful definition [38].

Our literature review identified the most relevant theories for understanding risk at the managerial and organizational levels, revealing diverse approaches and measurement methods. Early conceptualizations by Willett [39] and Knight [40] distinguished measurable risk from immeasurable uncertainty, using probability theory as a basis. Uncertainty, like risk, is multidimensional and can be viewed as a subjective state influenced by individual and situational factors [41]. Although risk and uncertainty are often differentiated in theory, many definitions in economics and management literature blur this line, treating them interchangeably (e.g., [42,43]). Research shows that managers seldom use probability calculus for risk assessment, relying instead on heuristics and context-driven preferences [37]. This reduces the relevance of Knight’s distinction between probable and indeterminate outcomes; therefore, we use these terms synonymously.

Risk is an integral part of strategic decision-making and is often featured in empirical strategic management research [36]. The term “strategic risk” was first coined by Roth in 1977 [44], but despite its importance, the literature still lacks a unified definition [45]. Broadly, strategic risk refers to the failure to achieve expected strategic outcomes and can be described as the risk encountered in pursuing strategic goals [46]. If a company’s actions involve strategy implementation, the associated risk is strategic [47]. Also, the conceptualization of strategic risk can sometimes be outweighed by its measurement. McConnell [48] argues that strategic risk is the most significant risk for any firm, yet difficult to measure. Typical measures include revenue volatility (e.g., [9,49]) or financial metrics (e.g., [50]).

Strategic risk occurs at senior management levels and relates to a company’s mission, long-term investments, and strategic planning. Research distinguishes between managerial and organizational risk, prompting questions about the role of managers in shaping organizational risk [51]. Palmer and Wiseman [3] equated strategic risk with managerial risk and suggested that only such risk influences the organization. Corporate risk often serves as a proxy for managerial risk [52]. Studies indicate that CEOs are the most influential in strategic risk decisions, impacting company outcomes [8,9]. Their strategic authority surpasses that of other senior executives [2], and their decisions directly affect stakeholders [13].

The role of top executives in firm performance has been explored through two main research streams [49,53]. The first, rooted in organization theory, suggests that organizational risk (measured by performance variability) results from both managerial risk-taking in CEOs’ strategic decisions and external factors (e.g., [3,8,54]). This approach indicates that while external factors dominate in the short term, CEOs’ strategic influence becomes more pronounced over time (e.g., [10,55–57]). The second stream focuses on specific high-risk investment decisions by CEOs that affect firm performance. Actions such as capital expenditures (CAPEX), R&D spending, and long-term debt are seen as indicators of strategic risk-taking (e.g., [7,25,58,59]).

Mergers and acquisitions (M&A) are another key area of high-risk strategic decision-making impacting firm outcomes [7,60]. Research, including PT [61,62] and Behavioral Theory of the Firm (e.g., [63–67]), supports this. Some studies even suggest CEOs may benefit from M&A regardless of the firm’s actual results (e.g., [68–71]). Both M&A and R&D investments are frequently used as proxies for assessing CEOs’ strategic risk-taking [4,53].

CEOs can make an error by taking excessive risks (Type I error) or being overly cautious (Type II error). AT and behavioral decision theories suggest different approaches to align CEO risk-taking with shareholder interests, using monitoring systems, incentives, or decision frameworks to mitigate biases [72]. The focus on the CEO as the main driver of strategic risk opens the field to psychological theories that examine risk perception, risk-taking, and risk-bearing.

Given the dual approach in studying strategic risk – managerial versus organizational – integrating these perspectives provides a more comprehensive understanding. Strategic managerial risk can be defined as the risk taken by senior executives, particularly CEOs, when making decisions that impact the organization’s strategic outcomes. This risk, driven by personal risk propensity and situational perceptions, includes both potential gains and losses that affect the executive’s personal stakes, such as job security and stock value. This definition highlights the importance of aligning external corporate incentives with managers’ individual traits, a field enriched by new behavioral theories [73]. The focus on both dispositional and situational factors helps deepen insights into CEO behavior, supported by psychological and motivational theories [20,74]. Understanding the circumstances surrounding these high-risk decisions is key to defining stakeholder-aligned risk levels and advancing strategic risk research.

### Regulatory focus

RFT, developed by Higgins [18,75], emphasizes the significance of aligning goals with an individual’s motivational orientation, which can enhance satisfaction and overall well-being [76,77]. RFT falls within the domain of cognitive psychology, viewing personality as a system of personal knowledge that integrates and processes information to guide actions [78]. The theory expands on Self-Discrepancy Theory [79], distinguishing between the “ideal self” (aspirations and desires) and the “ought self” (duties and responsibilities). While alignment with the “ideal self” results in high-engagement positive emotions like joy, alignment with the “ought self” yields low-engagement emotions such as calmness. Discrepancies can trigger negative emotions such as sadness or anxiety [80,81].

RFT outlines two primary motivational orientations: promotion focus and prevention focus [28,82]. Promotion-focused individuals strive for growth and achievement, perceiving risks as opportunities and viewing the status quo as an undesirable state of “no-gain” [83]. They tend to use ambitious, creative strategies aimed at progress [84]. In contrast, prevention-focused individuals prioritize safety, responsibility, and the avoidance of negative outcomes, perceiving the maintenance of the status quo as a positive “no-loss” outcome [85]. Their strategies are more cautious and defensive, aiming to protect resources and ensure stability [86–88].

Unlike Self-Discrepancy Theory, which considers “ideal” and “ought” standards as fixed personality traits, RFT treats promotion and prevention orientations as responsive to context. An individual can display both strong or mixed levels of each orientation, and RFT also accounts for situationally induced motivational states [89]. The theory extends to three levels of abstraction: system, strategy, and tactic, with tactical behaviors determined by immediate situational pressures [90]. Risky and conservative tactics are employed based on whether individuals are in a gain or loss domain, which influences their strategic choices [21,91,92].

Promotion-focused individuals in a gain domain may adopt conservative tactics after achieving sufficient progress, while prevention-focused individuals may choose risky tactics in loss domains to restore stability [21]. This nuanced approach, distinct from the conventional loss aversion described in PT, reveals that both orientations can display varied risk-taking behaviors within the same domain [79,93]. Research shows that prevention-focused individuals generally employ conservative strategies to maintain the status quo but may pivot to risky strategies when faced with potential loss [21].

RFT has gained prominence in management research as a key motivational trait affecting decision-making and strategic actions [20]. It provides a framework for understanding how CEOs evaluate strategic options and make decisions [80]. Unlike cognitive PT, RFT emphasizes goal pursuit and behavior mediation, offering deeper insights into strategic decision-making [79]. Studies highlight that compensation structures, particularly variable pay, can influence how regulatory focus translates into risk-taking behavior [59,94]. RFT posits that such external motivational mechanisms should shape managerial risk indirectly, contrary to the direct effects suggested by AT [15].

Promotion-focused individuals are driven by ambition and aim for growth and achievement [95], pursuing actions that align with desirable or ideal end-states. Typically, they exhibit greater creativity and a propensity to consider a wider range of options when making decisions [86,91,96]. Strong promotion focus can also lead to taking multiple actions, some of which may ultimately be flawed [18]. Such individuals often execute more options (occasionally without thorough analysis) rather than opt for inaction. Consequently, promotion-focused individuals are more susceptible to committing Type I errors as they strive not to miss potential opportunities [18], which can result in inefficient resource allocation. High promotion focus is also associated with a profit-oriented mindset [20], a greater tendency to make bold strategic decisions [97,98], and a willingness to invest resources in uncertain ventures. This leads to the following hypothesis:

**Hypothesis 1 (H1). CEO’s promotion focus positively affects strategic risk-taking.**

Prevention focus, on the other hand, centers on avoiding negative outcomes [95] and is embedded in the regulation of behaviors tied to duties and responsibilities [81]. Prevention-focused individuals aim to avoid discrepancies from desired goals and therefore act to mitigate potential threats or dangers that could impede their goal attainment [99]. Guided by a sense of duty [81,95,100], such individuals exercise due diligence [86,88], are generally less receptive to change [88], and demonstrate a tendency for repetitive patterns and considering fewer alternatives in problem-solving [99]. Prevention focus is linked to maintaining the status quo [85,101,102], risk aversion [103], careful planning, and vigilance [96].

While promotion focus is beneficial for opportunity-seeking, the caution associated with prevention focus aids in evaluating and selecting opportunities [86]. This caution and its related risk aversion can affect how prevention-focused CEOs perceive risky decisions and their potential impact on firm profit or loss. Research indicates that prevention focus has a negative association with exploratory actions but does not impact exploitative activities [104]. Unlike their promotion-focused counterparts, prevention-focused CEOs tend to explore fewer options [91]. Studies also show that prevention focus is associated with loss avoidance and the protection of limited resources [105], achievable through reduced spending on risky ventures. A strong prevention focus in a CEO may thus result in resource protection and an aversion to increasing expenditures on risky strategic initiatives to safeguard the company’s assets. This leads to the second hypothesis:

**H2. CEO’s prevention focus negatively affects strategic risk-taking.**

### Managerial incentives in the continental model of corporate governance

A corporate governance system encompasses the legal, institutional, and cultural framework within a country, influencing how stakeholders such as managers, shareholders, employees, and others shape managerial decision-making [106]. The connection between governance and national context results in distinct systems, even within similar overarching models [107,108]. While much of the literature has focused on Anglo-Saxon systems, there is an increasing emphasis on understanding governance in diverse geographic and institutional settings [32,109]. Ownership structures, executive compensation, and managerial motivations vary significantly across governance models, impacting strategic risk management [110].

In this context, an interesting case is Poland due to the relatively recent development of its corporate governance system, which started to take form during the economic transformation of the late 1980s. Polish companies, like those in other continental European nations, often exhibit high ownership concentration [111]. Most public firms are controlled by dominant shareholders, resulting in agency conflicts mainly between majority and minority shareholders, rather than between management and shareholders, as seen in Anglo-Saxon models [112]. The limited representation of minority shareholder interests is a notable issue in Polish governance [113].

The Warsaw Stock Exchange (WSE), reestablished in 1991, has been central in shaping corporate governance in Poland and is now the largest exchange in Central and Eastern Europe. By 2020, 89% of the 439 companies listed on the WSE’s main market were Polish, and 62.5% had financial institutions as shareholders [114]. Many of these companies are directly managed by their owners; family businesses make up 20.9%, while 32.7% include a board member who is also a shareholder. About 40% of the WIG index consists of companies with majority shareholders, showing limited separation between ownership and control [115].

Poland’s corporate governance is often seen as a hybrid model that combines elements of both continental European and Anglo-Saxon systems [116]. It features a dual-board structure, with management and supervisory boards functioning independently. The supervisory board appoints and oversees management board members and sets their compensation but relies on the management board for information [117]. This interdependence can sometimes result in trust issues and limited cooperation. Unlike the German model, Poland’s governance does not mandate employee participation in company management, a difference that distinguishes it from other Central European countries such as the Czech Republic and Hungary [118].

Anglo-Saxon influences are also evident through the Good Practices of WSE Listed Companies, introduced in 2002 and updated in 2021. These practices align with OECD principles, promoting shareholder equality and transparency but operate under a “comply or explain” system, which has led to challenges in enforcement, especially regarding board independence [119,120].

CEO compensation in Poland is similar to other countries, incorporating fixed compensation, annual bonuses tied to financial and strategic performance, interim performance bonuses, stock options, and non-cash perks such as health insurance and company cars. Equity-based incentives were introduced in the 1990s but remain underregulated, limiting their widespread use [121]. Survey data confirm that long-term incentive plans are offered by a minority of companies and typically apply only to selected board members, with most firms relying primarily on fixed pay and annual bonuses [23,122]. The design of these programs can vary significantly; for instance, stock options may not dilute ownership as participants typically do not gain voting rights, and strike prices can be set well below market value, decoupling them from firm performance [123,124]. Unlike standardized U.S. practices, Polish incentive programs are often tailored to company needs and stakeholder interests: conditions are often conservative, with strike prices set below market value and vesting contingent only on continued employment. Many plans lack meaningful performance thresholds and serve primarily as retention tools or deferred bonuses, rather than mechanisms to stimulate entrepreneurial risk-taking [124,125]. As a result, these instruments are widely perceived – by both managers and the market – as supplementary fixed pay with limited motivational value. Consequently, this study focuses on annual bonuses, and their role in influencing strategic risk-taking within Polish companies. Historically, the actual value of long-term instruments was difficult to isolate – it was often reported together with annual bonuses. As a result, the present analysis treats the bonus primarily as a cash-based component, although it may include equity elements (e.g., exercised warrants).

Regulatory focus can influence strategic managerial risk-taking, yet the applied incentive mechanisms can alter these relationships. Considering the distinctions between promotion and prevention focus, different forms of compensation may shape how regulatory focus translates to increased risk-taking. It is established that variability in compensation impacts risk-related behaviors [59], so CEOs with varying risk preferences may respond differently to incentive-based pay. The efficacy of compensation structures may be linked to individual motivations [94], and the perception of variable pay can shift depending on the leader’s regulatory orientation. The type of compensation, potentially more than its amount, can shape the behavior of CEOs with differing regulatory foci [126].

Incentive compensation for CEOs may moderate the link between regulatory focus and strategic decision-making. Incentive-based pay aims to influence CEO behavior and is associated with desirable outcomes such as knowledge-sharing within the firm [127], improved risk management [128], and greater firm engagement in research and development [129]. In fact, CEO bonuses, a form of incentive pay, have been particularly effective in explaining innovation-driven behaviors and outcomes [129] and have long been seen as a tool leveraged by boards to shape executive conduct [130,131].

Incentive compensation can place CEOs in situations of potential gain or loss. As Sanders and Hambrick [25] argue, stock options might push CEOs to “swing for the fences” to maximize potential gains, but they may also act defensively to safeguard these incentives, altering their effectiveness. Moreover, CEOs seeking immediate benefits might make short-sighted decisions to secure personal gains, sacrificing investments in long-term strategies that would bolster sustained profitability [132–134]. This behavior is especially relevant for prevention-focused executives who act to secure benefits and maximize wealth for both their firms and themselves. Reduced expenditure on high-risk strategies could lead to short-term positive impacts on incentive compensation value through cost savings and short-term earnings. Thus, significant incentive pay may prompt promotion-focused CEOs to cut spending on riskier initiatives in pursuit of short-term financial gains. Consequently, higher incentive compensation could negatively moderate the relationship between promotion focus and strategic risk-taking. These considerations lead to the following hypothesis:

**H3. Granting of annual bonus to CEO negatively moderates the relationship between promotion focus and strategic managerial risk, implying that the positive association between CEO’s promotion focus and strategic managerial risk weakens as annual bonus increases.**

Incentive compensation can also impact the link between prevention focus and strategic risk-taking. Individuals with a prevention focus aim to avoid actions that might create barriers to goal attainment [99], making them less likely to engage in behavior that introduces new uncertainties. Furthermore, prevention focus is associated with a heightened sense of duty and responsibility [81,95,135], which can reinforce adherence to performance criteria tied to incentive pay.

To motivate CEOs, supervisory boards structure compensation to encourage targeted behaviors. Prevention-focused CEOs are likely to align their behavior with board expectations to avoid forfeiting incentive compensation, even when such behavior diverges from their natural inclinations. Incentive pay can thus be employed to nudge prevention-focused individuals toward risk-taking. As outlined in hypothesis 4, higher annual bonus would positively moderate the relationship between prevention focus and strategic risk-taking:

**H4. Granting of annual bonus to CEO positively moderates the relationship between prevention focus and strategic managerial risk, implying that the negative association between CEO’s prevention focus and strategic managerial risk weakens as annual bonus increases.**

It is also crucial to consider the effect of fixed compensation on the relationship between CEOs’ regulatory focus and their strategic risk-taking. Fixed pay is considered steady and generally guaranteed as long as the CEO holds their position [136]. Unlike incentive pay, fixed compensation is associated with risk aversion [25,60,126,137], whereas incentive pay is seen as a remedy for a lack of bold decision-making. Given that regulatory focus influences an individual’s action orientation, understanding how fixed pay affects this relationship in top executives is essential. Fixed compensation is unique compared to bonuses as it is more likely perceived as “core” pay supporting a CEO’s standard of living [59]. Fixed pay ensures a level of financial stability, making the potential loss of this income (e.g., CEO dismissal) more impactful on the executive’s personal life than the loss of other forms of compensation.

Because promotion focus generally aligns with proactive behavior [81,88], it can be expected that promotion-focused CEOs would show a positive association with strategic risk-taking (Hypothesis 1). However, this relationship may be influenced by other factors. Fixed pay provides promotion-focused CEOs with a sense of stability that supports self-regulation in line with their natural tendencies. Higher fixed compensation ensures a stable income stream, allowing executives to make growth-oriented decisions [88], maximize firm value [18], and explore new opportunities [87]. Consequently, a higher level of fixed pay could “free” promotion-focused CEOs to pursue decisions that build future capabilities through new investments. This stable financial position may strengthen the natural tendency of promotion-focused CEOs to pursue growth via riskier strategies, leading to the following hypothesis:

**H5. CEO’s fixed compensation positively moderates the relationship between promotion focus and strategic managerial risk, implying that the positive association between CEO’s promotion focus and strategic managerial risk strengthens as fixed compensation increases.**

On the other hand, prevention-focused CEOs may respond differently to substantial levels of fixed income compared to their promotion-focused counterparts. Fixed income may be forfeited if the CEO is dismissed, creating a potential loss scenario that encourages risk-averse behaviors to safeguard their position and salary [136]. The threat of income loss is particularly relevant for prevention-focused actions, which are inherently risk-averse and geared toward avoiding value loss, including personal compensation. Higher fixed pay puts CEOs in a potential loss situation where poor firm performance could negatively impact their personal finances. CEOs seeking to protect their income may view potentially risky actions as threats to job security and associated salary. For highly paid prevention-focused CEOs, risk-averse tendencies such as protecting limited resources [105], minimizing risk [97], and avoiding potential losses (even at the cost of missed opportunities [18]) could be more pronounced than for prevention-focused CEOs with lower fixed pay. These CEOs may engage in behaviors aimed at safeguarding their stable income, such as avoiding new risky investments and cutting current expenditures to conserve resources, fostering cautious and potentially short-sighted actions. Based on the above considerations, the following hypothesis is formulated:

**H6. CEO’s fixed compensation negatively moderates the relationship between prevention focus and strategic managerial risk, implying that the negative association between CEO’s prevention focus and strategic managerial risk strengthens as fixed compensation increases.**

In summary, our theoretical model combines insights from the BAM [16], UET [10], and PT [17], enriched by the motivational framework of RFT [18]. This integration offers a more holistic perspective on CEO risk-taking by positioning regulatory focus as a key moderator in how executives interpret and act upon performance-contingent incentives. Our contribution lies in showing that motivational orientation is not merely a background trait, but a theoretically meaningful variable that interacts with governance mechanisms to produce observable strategic outcomes.

## Materials and methods

The considerations outlined in this study have culminated in the final form of a conceptual model for strategic managerial risk, encompassing both individual and situational factors. This model was developed through an analysis of the theoretical framework of corporate governance and theories of decision-making under uncertainty. Despite its multidisciplinary nature, the model is primarily grounded in strategic management. A graphical representation of the model is provided in S1 Fig.

The model substantially incorporates BAM [16,138]. Its added value lies in integrating variables from Higgins’s [18] psychological RFT. By synthesizing RFT with BAM, it can be concluded that motivational policy directed at top executives acts as a moderating factor between the independent variable, i.e. CEOs’ regulatory focus, and the dependent variable, which represents the risky ventures undertaken by the CEO on behalf of the firm [20].

Empirical research in management frequently relies on cross-sectional data, offering a snapshot of observations across various entities at a single point in time. In contrast, this study utilized panel data, capturing dependent, independent, and control variables for each entity across multiple time periods. This approach enabled longitudinal analysis, conducted using STATA for this purpose.

The data that support the findings of this study are openly available in Open Science Framework: https://osf.io/x3tak/

### Sample

The sample was selected from an initial pool of 429 companies listed on the Warsaw Stock Exchange (WSE), as identified in the Notoria database as of August 2023. The primary selection criterion was the availability of longitudinal data covering CEO communications (letters to shareholders), compensation, and firm-level performance indicators.

### CEO compensation and communication data

CEO compensation data, including base salaries and annual bonuses between 2011 and 2020, were extracted from the Refinitiv Eikon database and classified by industry. These data were cross-validated with financial statements, management reports, and public announcements available through Notoria. Notably, firms varied in how they disclosed bonus compensation (e.g., cash, subscription warrants, phantom shares, stocks and stock option grants [23,139]), which was carefully harmonized during the data preprocessing phase. It is worth noting that in company reports, annual bonuses were sometimes presented as cash-based and sometimes in the form of equity instruments. In this study, these differences were standardized. However, this means that the “bonus” variable encompasses various forms of compensation, not just pure cash.

CEO letters to shareholders were retrieved from 820 management reports accessed via the Infostrefa portal. These texts formed the basis for the content analysis method used to identify and measure each CEO’s regulatory focus. Due to significant data gaps in both this and other available sources, the final sample includes 82 publicly listed companies for which complete CEO communication and compensation data were available.

The companies included in the final analytic sample spanned 11 sectors of the Polish economy. The most represented industries were Construction and Real Estate (22%) and Finance and Insurance (21%), followed by Heavy Manufacturing and Media and Communication (each at 11%). Light Manufacturing, Health and Services, and IT/Technology accounted for approximately 9% and 8.5%, respectively. Other sectors such as Agriculture and Food (6%), Energy and Utilities (5%), and Trade and Retail (2.4%) were less frequently represented. Only one firm (1.2%) operated in the Transport and Logistics sector. These figures indicate a heterogeneous but somewhat finance- and real-estate-heavy sample, reflecting the structural composition of firms with accessible CEO-level data during the study period. The full sectoral breakdown is provided in S1 Table.

### Variables

#### CEO’s promotion focus and prevention focus.

Management literature emphasizes that psychological traits play a key role in shaping firm strategy and performance through their influence on managerial risk-taking tendencies [7]. One way to mitigate this issue is the use of content analysis – a method long established in sociology and increasingly applied in management sciences [140,141]. This technique represents a broad class of methods situated at the intersection of qualitative and quantitative traditions [141], and is recognized as valuable for uncovering consistent and meaningful insights from texts, such as the words, phrases, and linguistic patterns used by individuals in statements, organizational narratives, or other communication formats [142]. Emerging technologies that generate and archive digital traces provide researchers with innovative and extensive datasets that reflect real behaviors and can be collected and analyzed efficiently with modern tools [143].

Content analysis offers several methodological advantages over other research approaches. Most importantly for management research, it provides a replicable means of accessing deep individual-level structures. As such, content analysis is particularly suitable for examining complex and often elusive constructs, such as managerial cognition, due to its unobtrusive nature. This unobtrusiveness is especially relevant when studying senior executives, for whom access to direct data is typically limited [144]. Furthermore, the availability of standardized corporate documents over time (e.g., annual reports or industry publications) enables the implementation of longitudinal research designs targeting top managers [141].

Computer-aided text analysis (CATA) is a form of content analysis that enables researchers to operationalize constructs by transforming text into quantitative data based on word frequencies. In our study, we used CEO letters to shareholders to measure regulatory focus. These letters serve as rich corporate narratives that employ a wide and nuanced vocabulary, offering a platform for directing attention, highlighting significant developments, and shaping external perceptions. CEOs often use these letters to discuss organizational activities, events, and performance. As such, they are saturated with both rational and emotional linguistic elements, reflecting the CEO’s deliberate editorial framing [145].

To ensure semantic and lexical accuracy, the letters were digitized using ABBYY FineReader, converted to editable DOCX format, and manually corrected for OCR errors. The texts were then analyzed using the LIWC2015 software. Regulatory focus scores were calculated as the proportion of promotion- or prevention-related words to total word count, following the approach used by Murthy et al. [146]. This method is consistent with prior research that infers motivational dispositions from managerial discourse [20,145].

For Polish-language letters, we applied a validated dictionary developed by Marszałek, Miązek, and Roczniewska [147], while English-language letters were processed using the regulatory focus dictionary by Gamache et al. [20]. To date, no prior studies employing regulatory focus variables have offered a validated LIWC-based dictionary in the Polish language. This adaptation of the regulatory focus LIWC dictionary from Gamache et al. [20] was validated through a series of rigorously designed experimental and correlational studies. Collectively, these studies demonstrated the dictionary’s robustness and provided evidence for its content, discriminant, and convergent validity. The findings confirmed that the dictionary reliably identifies regulatory focus both in response to situational stimuli and as a stable individual characteristic [147], making it a suitable tool for research conducted in Polish.

The most contested assumption in content analysis is that the meaning of a message reflects the sender’s genuine intentions and is free from distortion, understatement, or overinterpretation. We acknowledge that shareholder letters are public-facing documents and may undergo input from investor relations or corporate communications teams. Therefore, the regulatory focus scores derived from these texts should be interpreted as indicators of the CEO’s *externally communicated* motivational emphasis rather than their internal cognitive state. This limitation is explicitly addressed in the *Discussion* section.

Nonetheless, research suggests that CEOs typically devote substantial time to drafting the main body of these letters, editing extensively, and embedding their own distinct voice [148,149]. This is especially relevant given that the CEO who signs the document is legally accountable for its content before financial regulators and socially responsible to a range of stakeholders [150]. CEO letters have also been used as a data source for constructing executives’ strategic schemas [151]. Numerous studies have confirmed the reliability of using content analysis for CEO letters, establishing the method as valid and credible (e.g., [152–154]). Criticisms of this approach should also account for its key advantage in examining psychological characteristics of executives: it provides a discrete, consistently available, and annually repeated metric that allows for longitudinal analysis [155]. Despite certain limitations, this approach likely offers a more accurate and consistent representation of the CEO’s outlook than self-reported measures subject to non-response bias [156]. For these reasons, in the present study we adopted validated Polish promotion and prevention dictionaries to analyze the motivational tone of CEO communications.

#### Strategic managerial risk.

The dependent variable in this model is strategic managerial risk, defined as the decision-making by top-level executives (particularly the CEO) that influences a company’s strategic outcomes (risk-taking). This is shaped by individual risk-taking tendencies (risk propensity) and the perceived level of risk in specific situations (risk perception), which poses personal risk exposure for the CEO (risk bearing). Such exposure carries both the potential for growth and the danger of losses, including declining stock prices or job termination.

Strategic managerial risk is commonly assessed through composite metrics that reflect risky decision-making. Related research has examined managerial behavior, CEO regulatory focus, acquisition activity, strategic actions, and firm performance, including the studies by Chng et al. [157], Gamache et al. [20], Seo et al. [158], Wallace et al. [159], and Wang G. et al. [160]. It’s essential to note that these measures do not equate to organizational risk but rather evaluate the risk inherent in strategic decision-making. The method involves aggregating multiple variables that approximate strategic risk, incorporating strategic options like diversification, international expansion, mergers and acquisitions, and financial leverage (e.g., [20]).

A widely tested approach is to use an indicator formed from the natural logarithm of the sum of long-term debt, R&D expenditures, and capital expenditures (CAPEX) [5,73,138,161–167]. R&D expenditures are operationalized as annual spending on research and development. CAPEX represents capital investments in tangible fixed assets. Long-term debt refers to obligations with maturities exceeding one year as recorded on the balance sheet (e.g., [60,73,138]). This study adopts this approach. The strategic managerial risk variable was constructed as an aggregate of R&D expenditures, total long-term debt, and capital investments, with data sourced from the EIKON database and company financial statements. The logarithmic index of these variables was derived from Refinitiv Eikon data: R&D expenditures (AING – Research & Development Costs, Gross) as reported on the balance sheet, total long-term debt (LTTD – Total Long-Term Debt) as recorded on the balance sheet, and CAPEX (Capital Expenditures) from the standardized cash flow statement.

While our strategic risk-taking variable reflects firm-level resource allocation under uncertainty via R&D intensity, CAPEX, and long-term debt [5,73,138,161–167], we acknowledge this approach does not distinguish between visionary, calculated investments and imprudent risk-taking [168]. For instance, high R&D intensity may signal innovation or reflect inefficient resource use. Similarly, leveraging via debt could indicate strategic expansion or excessive risk. Therefore, our measure should be interpreted as reflecting the intensity of strategic risk engagement rather than its normative quality or success. Importantly, our operationalization captures ex ante strategic commitments made under uncertainty, without assessing whether these decisions ultimately led to favorable or unfavorable ex post outcomes.

#### Moderating role of CEO’s compensation and incentives.

The model incorporates moderating variables related to compensation systems, which can influence the relationship between managers’ regulatory focus and their strategic risk-taking behavior. Numerous studies have examined how CEO compensation and governance mechanisms affect engagement in risky strategic decisions [25,60,169–172]. This research uses the variable incentive compensation, operationalized as CEO bonuses. Annual bonuses inherently carry a higher degree of risk and variability [59]. Additionally, fixed income was included as a second moderating variable, defined operationally as base salary [73].

Cash compensation, encompassing base salary and bonuses, is likely accounted for by CEOs in their current wealth assessments. Consequently, reductions in this compensation are perceived as losses [16]. Furthermore, this form of income would be forfeited in the event of the CEO’s termination. Given that managerial risk-bearing is negatively associated with organizational risk-taking, incentivizing managers with performance-based bonuses can mitigate aversion to uncertain outcomes [26]. To achieve a normal distribution of the data, the natural logarithm was applied to both measures [173].

#### Control variables.

The study includes control variables that may influence strategic managerial risk at both the individual (CEO-level) and organizational levels. Most of the control variables were obtained from the Refinitiv Eikon database, including: (1) CEO tenure within the company, sourced from the same section as salary and annual bonus data; (2) CEO ownership, retrieved from the Shareholders Report; (3) firm size, measured as the natural logarithm of total revenue (RTLR – Total Revenue) as reported in the income statement; (4) firm performance (ROA), calculated as the net income (NINC – Net Income) divided by total assets (ATOT – Total Assets) from the balance sheet.

CEOs’ decision-making can be affected by individual demographic factors [174–176]. Finkelstein and Hambrick [177] argue that the length of a CEO’s tenure significantly influences their willingness to take risks and act proactively, necessitating its inclusion as a control variable [178]. CEO tenure was operationalized as the number of years the executive has held the CEO position in the given organization [163,179].

In line with prior research, a CEO’s ownership (commonly associated with decision-making power and risk-taking) is also considered an important control variable [166]. It was measured as the ratio of shares owned by the CEO to the total number of shares issued by the company (e.g., [60,165,180–184]). Although some studies prefer to express ownership in monetary terms [70,179], we followed the standard proportional approach.

To further account for individual-level effects, we initially included CEO age and gender, as both factors have been linked to risk aversion [60,185]. For instance, CEOs nearing retirement age are less likely to engage in risky international acquisitions [7], and CEO age has been negatively associated with firm internationalization [186]. CEO age was calculated as the difference between the year of analysis and their birth year [166,173]. Gender was included as a binary variable, coded as “1” for male CEOs and “0” for female CEOs.

CEO succession was also included as a control variable, as leadership changes can significantly affect organizational direction and outcomes. CEO turnover was coded as “1” if a change occurred during the observation period and “0” otherwise [20,158,187].

At the firm level, we controlled for industry sector, firm size, and performance. Company size can influence risk-taking capacity, primarily through resource availability. The natural logarithm of revenue was used to capture this dimension. Revenue is commonly employed in the literature as a proxy for constructs related to company size [188]. It is also used as an operational measure for bureaucracy [189–191], complexity [191,192], resources [192–195], and legitimacy [70,192]. It is a widely accepted control variable in organizational studies.

Company performance was measured using return on assets (ROA), a widely used financial indicator that facilitates comparability across firms and studies. Prior research has suggested a negative relationship between performance and managerial risk-taking (e.g., [196–198]).

To address potential issues of time-series correlation, common in panel datasets where between-firm variation exceeds within-firm variation, we included year-fixed dummy variables for each year of the study period [199].

All independent and control variables were lagged by one year to ensure temporal separation between predictors and outcomes and to mitigate potential reverse causality [164].

### Method

To test our hypotheses, we used panel data models tailored to isolate within-firm variation over time. We employed fixed-effects (FE) panel regression models to estimate the relationship between CEO regulatory focus, compensation, and strategic managerial risk. This approach controls for unobserved, time-invariant firm-level heterogeneity, including organizational culture, sectoral norms, and governance environments, which could confound the effects of CEO traits on strategic outcomes. The Hausman specification test (χ² = 80.70, p < .001) confirmed the appropriateness of FE models over random-effects estimation [200].

Although variance inflation factor (VIF) diagnostics conducted in a pooled OLS model indicated acceptable levels of multicollinearity (all VIFs < 3.2), several variables were excluded from the FE models due to collinearity or lack of within-firm variation. Specifically, CEO gender, CEO succession, sector dummies, and CEO age were automatically dropped. CEO tenure, in contrast, was retained, as it varied over time and across firms.

To assess the robustness of our findings and the impact of excluded variables, we conducted supplementary analyses using random-effects (RE) and pooled OLS models with firm dummies. These alternative models allowed us to reintroduce time-invariant variables such as CEO gender, succession, and sector affiliation. The robustness checks confirmed that including these controls did not substantively alter the sign, magnitude, or significance of the main independent variables. CEO gender and sector were not statistically significant predictors of strategic risk-taking, and their inclusion did not change the overall interpretation of results. CEO tenure remained a marginally significant negative predictor of risk-taking, consistent with prior literature on managerial conservatism, but did not affect the core conclusions. Full estimation results comparing FE and RE models, including all control variables and selection corrections, are presented in S4 Table.

While FE models help mitigate bias from unobserved, time-invariant confounders, they do not fully resolve dynamic endogeneity or simultaneity issues. We considered alternative specifications such as instrumental variable (IV) estimation and system-GMM models, but the absence of valid and theoretically justified instruments limited their applicability. Moreover, our analytic goal was to capture within-firm variation over time – a setting where FE remains the most conservative and widely accepted approach in the management literature. Nonetheless, we acknowledge that residual endogeneity may persist and encourage future studies to employ alternative identification strategies, including IV or dynamic panel models, to more directly address potential simultaneity or reverse causality.

To address potential selection bias, we incorporated two Inverse Mills Ratios (IMRs) derived from Heckman two-step models. The first IMR corrects for selection into the analytic sample due to limited availability of CEO letters and compensation disclosures. The second IMR addresses potential bias arising from CEO turnover and changes in authorship of shareholder letters.

#### Correction for sample selection bias.

The limited availability of CEO letters and compensation data across the full WSE population raises the possibility of sample selection bias, particularly if the missing data are systematically related to CEO-level strategic behaviors. To address this issue, we applied a Heckman two-step correction procedure [201]. In the first step, we estimated a random-effects probit model predicting the likelihood of a firm being included in the final analytic sample, i.e., having complete data, based on firm-level variables theoretically unrelated to the dependent variable. These predictors included firm size (log of revenue), return on assets (ROA), industry sector, and CEO equity ownership. From this model, we derived the Inverse Mills Ratio (IMR_sample), which captures systematic differences between included and excluded firms. This IMR was then added as a regressor in the second-stage FE models. The IMR coefficient was statistically significant (p < .001), indicating that sample selection bias was present and effectively controlled for. Full model specifications and results are presented in S2 Table.

#### Correction for CEO succession bias.

In longitudinal panel data, internal validity may also be threatened by CEO turnover, which introduces unobserved heterogeneity. Since our regulatory focus variables are derived from annual letters and assigned to individual CEOs, changes in authorship across time can bias the measurement of motivational constructs. This issue is particularly relevant in fixed-effects models, where the dichotomous succession variable is absorbed by firm-specific effects and cannot be directly estimated. To mitigate this concern, we implemented a second Heckman two-step correction procedure [201], treating CEO succession as a potential source of non-random sample selection. In the first step, we estimated a probit model predicting the likelihood of CEO succession based on firm-level predictors associated with leadership transitions but not with CEO regulatory orientation. These included firm size (log revenue), financial performance (ROA), and CEO equity ownership. The resulting Inverse Mills Ratio (IMR_succession) captures latent selection effects related to CEO turnover and was added as a control variable in the second-stage FE regressions. This approach allows us to statistically adjust for potential structural bias caused by variation in CEO authorship, thereby preserving the validity of inferences about motivational constructs in a firm-level panel context. The full specification and results of the succession correction model are provided in S3 Table.

The IMR_succession coefficient was statistically significant in the main models (p < .001), indicating the presence of non-random CEO turnover that could bias motivational measurements if left uncorrected. This finding confirms that the applied correction effectively addressed selection effects related to CEO succession, enhancing the robustness of the estimated relationships.

## Results

Descriptive statistics and correlations (Tables 1 and 2) are consistent with theoretical expectations and confirm the internal validity of the dataset. CEOs in the sample were on average 58 years old, with a mean tenure of 11.8 years; 97% were male, reflecting the pronounced gender imbalance in top executive positions. Managerial ownership was modest (M = 7%, SD = 15%), but showed substantial variation across firms, suggesting heterogeneous levels of CEO influence.

**Table 1:** Descriptive statistics of variables.

No.	Variable	Observations	Mean	Std. Dev.	Min	Max
1	Promotion focus	786	1.66	0.76	0.00	5.08
2	Prevention focus	786	0.35	0.31	0.00	1.69
3	CEO fixed compensation (in thousand PLN)	589	934	829	4.00	5806
4	CEO bonus (in thousand PLN)	591	1027.53	2200	0.40	24241
5	Managerial strategic risk	818	17.87	4.00	0.00	24.37
6	CEO succession	820	0.52	0.50	0.00	1.00
7	Firm performance (ROA)	812	0.00	0.89	−24.32	0.54
8	CEO ownership	786	0.07	0.15	0.00	0.82
9	CEO gender	785	0.97	0.17	0.00	1.00
10	CEO age	284	57.65	8.13	38.00	79.00
11	CEO tenure (years)	758	11.80	7.54	1.00	33.00
12	Revenues (in thousand PLN)	818	3417825	11700000	139.00	120000000
13	Sector	820	4.72	2.49	1.00	11.00

Source: Own calculation.

**Table 2:** Pearson correlation coefficients.

No.	Variable	1	2	3	4	5	6	7	8	9	10	11	12
1	Promotion focus	1.00											
2	Prevention focus	−0.02	1.00										
3	CEO fixed compensation (in thousand PLN)	0.14**	0.10*	1.00									
4	CEO bonus (in thousand PLN)	−0.08	0.02	0.15**	1.00								
5	Managerial strategic risk	0.19**	0.02	0.52**	0.15**	1.00							
6	CEO succession	−0.08*	0.07	0.03	0.02	0.17**	1.00						
7	Firm performance (ROA)	0.09**	−0.02	−0.14**	0.15**	0.22**	−0.06	1.00					
8	CEO ownership	−0.19**	−0.10**	−0.18**	−0.09*	−0.21**	−0.33**	0.02	1.00				
9	CEO gender	0.12**	−0.02	0.01	0.03	0.04	−0.02	0.00	−0.05	1.00			
10	CEO age	−0.05	0.03	0.24**	0.06	0.08	−0.30**	−0.02	0.26**	0.02	1.00		
11	CEO tenure (years)	0.10**	−0.06	**−0.07**	**−0.05**	−0.19**	−0.74**	0.07*	0.32**	0.08*	0.32**	1.00	
12	Revenues (in thousand PLN)	−0.00	−0.03	0.13**	0.03	0.28**	0.11**	0.01	−0.05	0.03	0.10	−0.13*	1.00
13	Sector	0.08*	−0.13**	−0.09*	0.06	0.05	−0.02	0.04	−0.17**	0.14**	−0.05	−0.02	0.04

Note: **p < 0.01; * p < 0.05.

Source: Own calculation.

Compensation figures varied substantially: fixed salaries ranged from 4 thousand to over 5.8 million PLN (M = 934k, SD = 829k), while annual bonuses exhibited even greater dispersion ranging from 0.4 thousand to over 24.2 million PLN (M = 1.03 million, SD = 2.2 million), highlighting substantial differences in incentive structures. Strategic managerial risk, measured as a composite index of long-term debt, capital expenditures, and R&D intensity, exhibited moderate variability across firms (M = 17.9, SD = 4.0). Firm size, proxied by revenue, also varied substantially – from 139 thousand to 120 billion PLN (M = 3.42 billion, SD = 11.7 billion) – highlighting the structural diversity of the sampled companies.

Table 2 confirms that promotion and prevention focus were only weakly correlated (r = –0.02, ns), supporting their treatment as orthogonal constructs and justifying their simultaneous inclusion in regression models. Additional relationships worth noting include the strong positive correlation between managerial risk and CEO fixed compensation (r = 0.52, p < 0.01), suggesting that firms with more risk-taking CEOs tend to offer higher base pay. As expected, CEO tenure was strongly and negatively correlated with CEO succession (r = –0.74, p < 0.01). Moreover, CEO ownership was negatively associated with both promotion focus and strategic risk-taking, possibly indicating a more cautious orientation among owner-CEOs.

Next, we turn to the hypothesis tests. Model 1 in Table 3 presents the baseline fixed-effects regression including the compensation variables and control variables. The analysis reveals that CEO fixed compensation (β = –1.139, *p* < 0.001) is a strong negative predictor of strategic managerial risk. This finding aligns with behavioral agency theory, which posits that fixed, guaranteed compensation tends to dampen executives’ willingness to engage in risk-intensive strategic initiatives. In contrast, the annual bonus (β = 0.578, *p* < 0.001) is positively and significantly associated with strategic risk-taking, suggesting that performance-contingent incentives may encourage CEOs to pursue more aggressive or uncertain strategies.

**Table 3:** Regression analysis results.

Variable	Model 1	Model 2	Model 3	Model 4	Model 5
**β (SE)**	**β (SE)**	**β (SE)**	**β (SE)**	**β (SE)**
Promotion focus		0.695*** (0.063)	0.610*** (0.079)	0.643*** (0.066)	0.570*** (0.080)
Prevention focus		−0.212* (0.102)	−0.252* (0.111)	−0.272* (0.131)	−0.242* (0.12)
Promotion focus x CEO bonus			0.306* (0.138)		0.275* (0.108)
Prevention focus x CEO bonus			0.154* (0.062)		0.152* (0.062)
Promotion focus x CEO fixed compensation				−0.195** (0.075)	−0.190** (0.076)
Prevention focus x CEO fixed compensation				−0.159** (0.068)	−0.168** (0.068)
CEO fixed compensation (ln)	−1.139*** (0.159)	−2.445*** (0.173)	−2.434*** (0.173)	−2.448*** (0.172)	−2.445*** (0.173)
CEO bonus (ln)	0.578*** (0.119)	1.355*** (0.121)	1.084*** (0.174)	1.391*** (0.121)	1.132*** (0.174)
ROA	−0.461 (0.574)	−1.199** (0.513)	−1.280** (0.514)	−1.298** (0.511)	−1.386*** (0.512)
CEO ownership	0.194 (0.140)	−0.050 (0.123)	−0.072 (0.123)	−0.054 (0.122)	−0.077 (0.122)
CEO tenure	−0.086 (0.098)	−0.146* (0.085)	−0.132 (0.085)	−0.168** (0.085)	−0.154* (0.085)
Revenues (ln)	0.365 (0.858)	−1.560** (0.760)	−1.663** (0.763)	−1.786** (0.761)	−1.916** (0.765)
IMR sample	6.205** (2.429)	13.416*** (2.185)	13.408*** (2.179)	13.317*** (2.177)	13.297*** (2.171)
IMR succession	−7.494*** (0.765)	−16.314*** (0.966)	−16.459*** (0.976)	−16.627*** (0.967)	−16.808*** (0.979)
Constant	18.151*** (1.275)	15.236*** (1.988)	15.415*** (1.984)	15.608*** (1.982)	15.786*** (1.978)
Observations	573	573	573	573	573
Groups	80	80	80	80	80
*R2 within*	0.3982	0.5487	0.5532	0.5559	0.5603
*R2 between*	0.3961	0.1891	0.1852	0.1906	0.1864
*R2 overall*	0.3286	0.1287	0.1252	0.1296	0.126

Note: Standard errors in parentheses.

*** p < 0.001, ** p < 0.01, * p < 0.05.

Year 2011 is the reference category and omitted from the models. Year dummies (2012–2020) are included in all models unless excluded due to collinearity.

Time-invariant variables, such as CEO gender and succession status, are excluded from fixed-effects models due to collinearity or lack of within-firm variation. Sample- and succession-related selection biases were addressed using the Inverse Mills Ratio (IMR) derived from two-step Heckman correction models.

Source: Own calculation.

Other firm-level controls show mixed effects. Although firm performance (ROA) (β = –0.461, *p* = 0.26) and CEO ownership (β = 0.194, *p* = 0.17) are not statistically significant in this model, CEO tenure is negatively associated with risk (β = –0.086, *p* = 0.07), approaching significance. Similarly, firm size, measured as log-transformed revenues, shows a positive but non-significant effect (β = 0.365, *p* = 0.66), suggesting no robust size effect at this stage.

Importantly, both IMR variables addressing selection bias are statistically significant: the IMR from the sample selection model (β = 6.205, *p* = 0.01) and the IMR related to CEO succession (β = –7.494, *p* < 0.001), confirming that accounting for non-random sample inclusion and succession likelihood is crucial for consistent estimation. This baseline model explains approximately 40% of the within-firm variance in strategic risk (R^2^ within = 0.398), providing a solid foundation for subsequent models that incorporate CEO psychological traits and interaction effects.

Adding CEO regulatory focus in Model 2 confirmed the directional hypotheses. CEOs with a promotion focus demonstrated significantly higher strategic risk-taking (β = 0.695, *p* < 0.001), while those with a prevention focus exhibited significantly lower levels of risk (β = –0.212, *p* = 0.04), thus supporting Hypotheses 1 and 2. These findings are consistent with regulatory focus theory: promotion-oriented CEOs are more inclined toward strategic initiatives involving uncertainty and growth, whereas prevention-oriented CEOs tend to avoid actions that could jeopardize stability or control.

The inclusion of individual-level psychological traits meaningfully improved model fit. The within-group R^2^ increased from 0.398 in Model 1 to 0.549 in Model 2, highlighting that CEOs’ motivational orientations help explain time-varying fluctuations in strategic risk within firms. This reinforces the value of integrating upper echelons theory with regulatory focus theory to better understand executive behavior.

Importantly, the effects of compensation and other controls remained robust. Both CEO bonus (β = 1.355, *p* < 0.001) and fixed salary (β = –2.445, *p* < 0.001) retained their strong and opposing associations with risk-taking, while ROA had a significant negative effect (β = –1.199, *p* = 0.02), suggesting that stronger performance may reduce the perceived need for risky strategic action. CEO tenure was marginally significant (β = –0.146, *p* = 0.08), indicating that more experienced CEOs may become more cautious over time.

Additionally, the Inverse Mills Ratios (IMR) from the Heckman correction remained highly significant (sample IMR: β = 13.416, *p* < 0.001; succession IMR: β = –16.314, *p* < 0.001), reaffirming the necessity of correcting for selection biases in CEO panel data. These effects confirm that the sample is not randomly composed, and succession dynamics substantially shape observable CEO behavior.

Models 3 and 4 tested the moderating effects of compensation structure on the relationship between regulatory focus and strategic risk. In Model 3, which includes interaction terms between regulatory focus and CEO bonus, both interactions were statistically significant and positive. The interaction between promotion focus and bonus was significant (β = 0.306, *p* = 0.02), contrary to Hypothesis 3, which had anticipated a mitigating effect. Instead, the results suggest that promotion-focused CEOs become even more risk-seeking when incentivized with high bonuses, potentially due to a “regulatory fit” between intrinsic motivations and extrinsic rewards.

Similarly, the interaction between prevention focus and bonus was also positive and significant (β = 0.154, *p* = 0.02), supporting Hypothesis 4. This indicates that even prevention-oriented CEOs, typically risk-averse, may be nudged toward riskier behavior when bonuses are at stake. These patterns are consistent with motivation-cognition alignment frameworks, where performance-contingent pay acts as an activating mechanism even for conservative decision-makers.

Model 4 replaces bonuses with fixed salary as the moderating variable and provides further insights into the dynamics of regulatory focus and risk. Both interaction terms were again statistically significant and, notably, negative. The interaction between promotion focus and fixed pay was significant (β = –0.195, *p* = 0.01), contradicting Hypothesis 5, which predicted a positive moderating effect. This suggests that guaranteed income dampens risk-taking even among promotion-focused CEOs, possibly by lowering the perceived urgency or marginal utility of bold strategic initiatives.

The interaction between prevention focus and fixed pay was also negative and significant (β = –0.159, *p* = 0.01), supporting Hypothesis 6. For prevention-focused CEOs, fixed salary appears to reinforce their inherent risk aversion, reducing their inclination to pursue uncertain strategies. These effects are visualized in S4 and S5 Figs, which show that higher fixed compensation attenuates the positive relationship between promotion focus and strategic managerial risk, while strengthening the negative relationship between prevention focus and strategic managerial risk.

Model 5 presents the fully specified model, incorporating all hypothesized main effects and interactions. The estimates remain consistent with previous models, confirming the robustness of the findings. All interactions are statistically significant and in the same direction as in Models 3 and 4: bonus interactions are positive (promotion × bonus: β = 0.275, *p* = 0.02; prevention × bonus: β = 0.152, *p* = 0.02), while fixed pay interactions are negative (promotion × fixed pay: β = –0.190, *p* = 0.01; prevention × fixed pay: β = –0.168, *p* = 0.01).

These results underscore that the effects of compensation structure on CEO risk-taking are not uniform, but contingent on individual motivational orientation. Bonuses appear to amplify risk-seeking behavior (especially when aligned with a promotion focus) whereas fixed compensation exerts a constraining influence across both motivational profiles.

The model’s explanatory power is the highest among all specifications, with R^2^ within = 0.560, confirming that the integration of psychological and structural factors offers the most complete account of strategic risk behavior. Notably, the strong and persistent significance of both IMR terms (sample IMR: β = 13.297, *p* < 0.001; succession IMR: β = –16.808, *p* < 0.001) again highlights the importance of correcting for non-random sample selection and succession dynamics in CEO-level analyses.

Although Hypotheses 3 and 5 were not supported in the expected direction, their results nonetheless yield theoretically meaningful insights. Specifically, the amplifying effect of bonuses for promotion-focused CEOs may reflect regulatory fit, where extrinsic rewards align with intrinsic motivations, leading to overconfidence and elevated risk engagement [21,202]. Conversely, the negative effect of fixed salary across motivational types may indicate that secure compensation activates loss aversion or reduces the perceived urgency to pursue strategic change [16].

This asymmetry suggests that risk-related behavior is not uniformly moderated by incentive type, but contingent on motivational orientation. The model’s explanatory power is strong but also points to the relevance of other omitted moderators, such as firm lifecycle stage or governance activism, that may condition these effects.

Together, the regression models and interaction plots (S2–S5 Figs) show that regulatory focus and compensation interact in nuanced and sometimes counterintuitive ways. The data suggest that promotion-focused CEOs are not necessarily moderated by bonuses in a risk-averse direction; rather, these incentives may reinforce their natural tendencies. Prevention-focused CEOs, in contrast, appear more malleable to external incentives, becoming more risk-seeking under high bonus pressure and more cautious under high fixed pay. These asymmetrical patterns underscore the need for boards to carefully consider the alignment of compensation systems with CEO psychological profiles.

This research confirmed the direct impact of CEOs’ regulatory focus on their strategic decision-making, which subsequently influences the firm’s strategic outcomes. As expected, promotion-focused CEOs were more inclined to take riskier decisions, thereby increasing the company’s strategic risk. On the other hand, prevention-focused CEOs adopted more cautious approaches to mitigate risk. These results align with previous studies, such as those by Gamache et al. [20], who measured strategic risk through the number and value of acquisitions, and Scoresby et al. [203], who associated it with R&D investments. They contribute to the expanding management literature that underscores the predictive power of regulatory focus as a key motivational trait among top executives.

The interaction effects of external motivational mechanisms – fixed compensation and annual bonuses – were particularly noteworthy. Salaries, which are not directly tied to short-term performance, and annual bonuses, contingent on meeting specific company targets, were analyzed. In all models, fixed compensation exhibited a negative relationship with strategic risk (β = –2.445, *p* < 0.001 in Model 5), while annual bonuses showed a positive relationship (β = 1.132, *p* < 0.001). These findings are consistent with research by Gamache et al. [20] and Scoresby et al. [203], and reinforce agency and behavioral theories advocating for performance-based incentives. This emphasizes the need for comprehensive motivational programs that incorporate varied components and suggests that high salaries (often seen as excessive) may not necessarily enhance company performance. This issue has been widely debated, especially in the U.S. after the global financial crisis, where CEO compensation was found to be disproportionate to company results, even in periods of severe financial stress [204–206]. In contrast, the pay gap between CEOs and employees in continental and Asian corporate models is generally narrower than in the U.S., where CEOs earn 265 times more than the average worker [24].

Concerning the moderating effect of annual bonuses, the findings did not support the hypothesis that higher bonuses would weaken the positive relationship between a CEO’s promotion focus and strategic risk-taking. It was hypothesized that significant incentive compensation might encourage promotion-focused CEOs to limit risky investments in pursuit of short-term financial gains. However, the results suggest that CEOs’ inherent motivational tendencies remain strong and are not easily altered by situational incentives. An interaction plot (S2 Fig) shows that the relationship between promotion focus and strategic risk is consistently positive, and is more pronounced at higher bonus levels (dashed line), indicating that substantial bonuses may amplify the inherent risk-taking tendencies of promotion-focused CEOs.

This result contradicts the assumption that financial rewards would encourage more conservative behavior in promotion-focused leaders. Instead, it supports the idea that individuals with a promotion focus are particularly sensitive to gain opportunities and take greater risks when such opportunities are emphasized [18,21]. Bonuses may thus act as accelerants, aligning situational incentives with dispositional preferences – a phenomenon consistent with regulatory fit theory and possibly overconfidence effects [202]. Boards should recognize that promotion-focused executives might interpret performance-contingent pay not as a constraint but as an invitation to pursue more aggressive strategic moves.

For prevention-focused CEOs, the moderating effect of bonuses on the relationship between regulatory focus and strategic risk-taking was positive, consistent with Hypothesis 4. As illustrated in S3 Fig, the negative relationship between prevention focus and strategic managerial risk is clearly visible under low bonus conditions (solid line). However, this relationship becomes non-significant under high bonus conditions (dashed line), where the slope is close to zero. This pattern indicates that performance-based bonuses can neutralize the risk-averse tendencies of prevention-focused CEOs, weakening the negative association between prevention focus and strategic risk-taking.

The final set of interaction effects examined the role of fixed compensation in moderating the relationship between CEOs’ regulatory focus and strategic managerial risk. The results revealed divergent patterns depending on motivational orientation.

For promotion-focused CEOs, the interaction between fixed salary and strategic risk-taking was negative and significant, contradicting Hypothesis 5. As shown in S4 Fig, the positive association between promotion focus and strategic risk is visible under low fixed compensation (solid line), but becomes nearly flat under high fixed compensation (dashed line). This suggests that guaranteed pay suppresses risk-taking tendencies even in executives predisposed toward bold, achievement-driven strategic action. Although initially counterintuitive, this finding aligns with motivational theories that emphasize how excessive security can blunt the behavioral activation associated with promotion focus. Lower fixed compensation may be perceived as a signal of performance-contingent pressure or income insecurity, thus activating goal-pursuit and risk-taking behavior among promotion-focused individuals. In contrast, high fixed salary may reduce this internal urgency, making executives more complacent or risk-averse. Rather than reinforcing boldness, fixed compensation may buffer it – particularly when motivational drivers are already high. This pattern may also reflect a nonlinear dynamic in which moderate levels of fixed pay enhance performance, but excessively high levels suppress behavioral initiative. Future research could test this possibility by modeling curvilinear or threshold effects.

In contrast, results for prevention-focused CEOs provided clear support for Hypothesis 6. As illustrated in S5 Fig, the negative association between prevention focus and strategic risk becomes stronger under high fixed pay (dashed line), compared to the relatively weak relationship observed under low fixed pay (solid line). This indicates that fixed compensation reinforces cautious tendencies, likely by increasing the perceived cost of failure or amplifying risk aversion.

Taken together, the findings suggest that fixed compensation functions as a psychological anchor: it tempers boldness in promotion-oriented CEOs and amplifies caution in prevention-focused ones. This asymmetry underscores the importance of aligning compensation design not only with organizational goals, but also with the underlying motivational orientation of the CEO.

These results confirm that external incentives do not operate uniformly across CEO motivational profiles. While performance-based bonuses appear to amplify strategic risk-taking in promotion-focused CEOs and neutralize risk aversion in prevention-focused ones, fixed salaries consistently suppress risk-taking, most strongly among prevention-oriented executives. The interaction plots support these interpretations and visually reinforce the asymmetric moderation patterns identified in the regression models.

## Discussion

This study developed an integrative theoretical framework combining Regulatory Focus Theory (RFT) with key perspectives from corporate governance research – specifically, Upper Echelons Theory (UET), the Behavioral Agency Model (BAM), and Prospect Theory (PT). The findings broadly confirmed our expectations while also revealing a complex pattern of interactions between CEO characteristics and compensation-based incentives. As predicted, CEOs with a strong promotion focus exhibited higher levels of strategic risk-taking, whereas prevention-focused CEOs demonstrated greater caution, avoiding risky decisions that could jeopardize goal attainment. Importantly, our analysis shows that these motivational dispositions did not operate in isolation – their effects were systematically moderated by compensation structures.

However, these moderating effects did not always conform to our initial hypotheses. Contrary to expectations, bonuses intensified risk-taking in promotion-focused CEOs (rejecting Hypothesis H3), while fixed salaries appeared to reduce risk-taking even in this group (rejecting H5). These unexpected outcomes offer new insight into motivational mechanisms at the top of the organization.

First, the observation that bonuses failed to “rein in” promotion-focused CEOs, but instead appeared to amplify their risk appetite, suggests a regulatory fit effect. When external incentives (financial bonuses) align with internal motivation (promotion-driven goal pursuit), they may reinforce rather than counteract existing behavioral tendencies. In other words, promotion-focused CEOs may have interpreted performance-based bonuses as validation of their risk-seeking strategies and as signals to continue or even escalate such behaviors.

This may also reflect an overconfidence mechanism, wherein leaders receiving high performance-based compensation perceive it as a reward for their vision and strategy, thereby fueling further bold decision-making [21,202]. We discussed this finding in light of regulatory fit theory and overconfidence literature, proposing that motivational congruence between internal dispositions and external incentives can magnify behavioral responses rather than neutralize them.

Second, the broadly consistent dampening effect of fixed salaries on risk-taking was equally noteworthy. High base pay may function as a safety anchor, offering financial comfort even for promotion-focused leaders, partially curbing their appetite for risky ventures. We interpret this pattern as evidence of loss aversion and a comfort zone effect: once a CEO is granted a secure, substantial income, they may become more inclined to protect this certainty from potential threats (e.g., job loss following failure) [16]. This mechanism appeared particularly pronounced among prevention-focused CEOs, which is unsurprising given their inherent loss sensitivity; but the effect was also statistically significant (albeit weaker) among promotion-focused CEOs. This suggests that even risk-tolerant leaders are responsive to stable income by exhibiting reduced behavioral dynamism, ultimately disconfirming Hypothesis H5.

In the literature, this phenomenon raises the possibility of a non-linear relationship: a moderately high salary may instill a sense of security conducive to investment activity (“I have a base, so I can take risks”), whereas exceeding a certain threshold may reduce motivation for additional risk-taking (“I’m already well-compensated, so why jeopardize that?”). Following Wiseman and Gómez-Mejía [16], we propose that executive pay effects may follow threshold or curvilinear patterns – a hypothesis worthy of further empirical testing.

A central insight from our study is therefore that common governance instruments (bonuses, salaries) generate non-neutral behavioral outcomes. Their impact is contingent on the stable characteristics of the individuals they target. This challenges traditional agency theory assumptions, which often treat executives as a homogeneous group responding predictably to financial stimuli. Our findings indicate that there are no universally “neutral” incentives: for example, a bonus might push one CEO into aggressive forward motion, while encouraging another to “catch up” and approach a desired risk level. The asymmetries we identified (bonuses energize different types of CEOs through different mechanisms; fixed pay affects them more uniformly) add a behavioral layer to agency theory and call for more nuanced incentive design.

This study makes several theoretical contributions. To avoid redundancy, we do not revisit full theoretical definitions here but instead focus on interpreting findings in light of these established perspectives [10,18,41]. First, we extend UET by demonstrating that stable motivational traits (promotion/prevention focus) exert a direct influence on CEOs’ strategic actions, beyond commonly examined demographic and experiential variables. This supports a growing view that psychological differences among top managers are critical, yet underexplored, determinants of strategic outcomes.

Second, we contribute to the BAM by showing empirically that CEO motivation conditions the effectiveness of incentive mechanisms. Compensation does not operate in a vacuum – its behavioral consequences vary based on whether a CEO is primarily gain-seeking or loss-averse. This enriches our understanding of the principal–agent relationship, emphasizing that both contract structure and agent psychology jointly shape outcomes.

Third, we integrate PT with RFT, illustrating that CEOs’ chronic promotion/prevention orientations influence how they frame and respond to gain–loss scenarios. For instance, a promotion-focused CEO may continue to pursue high-risk strategies even when high base pay creates a “potential loss” frame (which should discourage risk-taking under PT), suggesting that their achievement motivation outweighs loss-framing effects. Conversely, prevention-focused CEOs remained cautious even when bonuses created a “potential gain” scenario, although the bonus attenuated their risk aversion. Such patterns advance PT by incorporating stable individual differences in gain/loss sensitivity.

The practical implications of these findings are substantial. Corporate boards and policymakers should recognize that compensation structures interact with executive psychology in complex and asymmetric ways. For boards, the key message is: know your CEO. Effective incentive design requires identifying whether the leader is inherently risk-tolerant or overly cautious. Motivational profiling (e.g., regulatory focus, risk tolerance assessments) should become a routine element in CEO selection and evaluation. Managerial contracts should be tailored, not one-size-fits-all. For example, promotion-focused CEOs may require capped bonuses and stronger risk control mechanisms such as clawbacks or long-term incentive plans (LTIPs) that link rewards to sustained performance rather than short-term wins. In contrast, prevention-focused leaders may benefit from carefully designed performance-based bonuses, tied to strategic goals and monitored to avoid unintended operational risks.

Another practical implication concerns leadership team composition. If a CEO exhibits strong promotion focus, appointing a CFO or senior advisor with a more prevention-oriented profile may provide cognitive balance in decision-making. Prior research and practice both suggest that cognitively diverse teams are more effective in assessing risks (e.g., [207]). Our findings support this and highlight the value of psychological diversity in the C-suite.

On a broader level, our results have implications for compensation policy across governance systems. The 2008 financial crisis illustrated how poorly designed bonus schemes can fuel excessive risk-taking with catastrophic outcomes [208]. Analogous tendencies have also been observed in the tech sector. Founder-CEOs with high equity ownership and strong promotion focus have frequently undertaken “moonshot” R&D projects – characterized by high risk and uncertain returns – which exemplify the interaction between dispositional traits and high-powered incentives [209].

Our findings emphasize that this risk is especially acute when aggressive incentives are deployed in combination with visionary, unchecked CEOs. Hence, regulators and boards should implement safeguards such as non-financial performance conditions, bonus deferrals (to enforce long-term perspective), and hard caps on variable pay. The European Union has begun moving in this direction (e.g., bonus caps in banks, Shareholder Rights Directive II 2017/828 disclosure requirements), and our results provide additional rationale: transparency and pay control should account for the human factor. Practically, this could mean that remuneration reports would not only list figures but also offer a narrative justification for why the structure is appropriate, for example: “CEO X is oriented toward expansion, so strategic objectives were set as bonus triggers, with a maximum cap to protect against excessive risk”. Such narrative-driven disclosure would increase shareholder confidence and demonstrate proactive behavioral risk management.

In sum, our discussion underscores that the individual characteristics of decision-makers are critical to the behavioral outcomes of governance mechanisms. We introduce the concept of incentive–CEO asymmetry as a potential explanation for inconsistencies in the literature, e.g., why some studies find positive effects of stock options while others report negative outcomes (perhaps depending on the dominant CEO type in the sample). We advocate a shift from universalism toward people-centered governance, in which governance solutions are designed with specific individuals (and their behavioral responses) in mind.

### Limitations and future research directions

While this study has certain empirical limitations, its scope and findings open several avenues for future research. A key issue concerns the sample selection, particularly whether only 82 publicly listed companies on the Warsaw Stock Exchange (WSE) use annual bonuses for CEOs. Identifying firms linking CEO performance-based compensation to strategic risk-taking is challenging, since detailed remuneration disclosures were mandated in Poland only in 2020 [23]. For this reason, this study spans 2011–2020, and future research is needed to compile a complete list of such firms from years beyond that period.

Our sample excludes small and medium-sized enterprises (SMEs), focusing solely on publicly traded firms with available executive compensation and communication data. This introduces a selection bias, as SMEs often operate under different governance structures and risk dynamics [210]. While our findings are applicable to listed firms in Central Europe, they may not generalize to smaller or privately held companies. Future studies should explore whether the motivational mechanisms identified here replicate in SME contexts, where CEOs often hold ownership stakes and face less formalized incentive systems. Moreover, in SMEs, the personal ownership stake of CEOs often intensifies their exposure to strategic outcomes, which may either amplify or dampen the effects of dispositional traits such as regulatory focus.

Our study did not sufficiently account for sector-specific factors. While we initially included a control variable for industry sector, it was automatically excluded from the fixed-effects models due to collinearity. In the random-effects model (see S4 Table), sector was retained but did not show a statistically significant association with strategic managerial risk. Although our sample included 82 companies spanning 11 different sectors, its limited size prevents us from drawing reliable conclusions about the sectoral impact on CEO risk-taking behavior. While we account for firm-level fixed effects, which absorb time-invariant industry characteristics, we acknowledge that sectoral factors may still influence CEOs’ propensity for strategic risk. For instance, firms in banking or utilities face tighter regulatory environments compared to those in technology or manufacturing [211]. Due to sample size limitations, a finer-grained analysis of industry effects was not feasible here. Future research with larger, sector-diverse samples should further investigate how regulatory focus interacts with sector-specific risk norms. Such sectoral norms may further shape how CEOs cognitively frame strategic boldness as either a necessary innovation or a reckless deviation, depending on industry conventions.

One limitation of our approach is the measurement method of regulatory focus, which relies on sentiment- and frequency-based analysis of CEO letters to infer regulatory focus. While validated and widely used in upper echelons research [20], this method does not fully capture rhetorical nuance, irony, or contextual subtleties in language use [141,142]. Additionally, CEO letters may reflect collaborative input from investor relations departments. We therefore treat regulatory focus not as a direct measure of internal cognition, but as a publicly communicated motivational stance. Nonetheless, future studies might explore rhetorical layer analysis or discourse-based sentiment frameworks to address deeper linguistic nuance. Future research may benefit from triangulating this method with qualitative interviews, behavioral assessments, or experimental designs to improve construct validity and interpretive depth. While we acknowledge that linguistic sentiment analysis has limited capacity to fully capture the contextual subtleties of CEO motivation, its consistency and unobtrusiveness make it a valuable proxy – particularly when interpreted within a broader methodological framework that includes robustness checks and theoretical triangulation.

Another important limitation (though also a contextual contribution) is the focus on firms operating within the Polish corporate governance model. Its institutional specificities may influence how incentive systems function. For example, some Polish firms use equity-based compensation primarily as a retention tool rather than to encourage risk-taking. The structure and exercise conditions of stock options are critical. However, the transparency of executive remuneration disclosures has historically been inconsistent. It should be emphasized that only since 2020 have Polish companies been required to publish remuneration reports with a breakdown of equity instruments. This revealed that a significant portion of variable compensation components (around 44%) took the form of subscription warrants (an instrument functionally similar to stock options – see [212,213]). In earlier years, the value of these instruments was concealed under broader categories such as “annual bonus,” which made analysis more difficult [23,139]. Future research should assess how these compensation forms affect managerial decision-making.

There is also a gap in cross-national empirical studies on agency issues, dominated by U.S.-based theories. This study adapted a research model to the continental corporate context while drawing on U.S.-centric theoretical foundations, underscoring the need for more Europe-focused empirical research. It is a step forward but does not fully address this imbalance.

Complementary studies could explore European corporate models, focusing on agency issues, desired managerial risk behaviors from shareholders’ perspectives, and related incentive systems. These studies could examine individual countries or conduct international comparisons, supporting hypotheses tailored to the continental corporate model in strategic managerial risk.

As a robustness consideration, we also explored alternative strategic risk proxies, such as M&A activity and international expansion. Although comprehensive data were not available for all firms in the sample, post-hoc analyses revealed that firms engaging in M&A tended to score higher on our composite index. This suggests that our primary measure captures broader patterns of risk-oriented strategic behavior. This supports the validity of our primary indicator as a proxy capturing firms’ engagement in bold, forward-looking initiatives. Nevertheless, future research could explicitly incorporate additional indicators, including acquisition frequency, geographic diversification, or performance volatility, to develop more fine-grained operationalizations of CEO-driven strategic risk [129,130,134].

Future studies should examine boundary conditions that may shape the compensation-risk relationship. Institutional context may be particularly important – for instance, stakeholder-oriented firms in coordinated market economies may respond differently than shareholder-driven firms in liberal economies. In Poland, where ownership is often concentrated and board independence varies, situational moderation of motivational traits may be weaker or more nonlinear [113,119,120]. Understanding how national governance models and cultural values affect incentive salience could refine theoretical models and guide compensation policy tailoring. Cross-national research may also illuminate how stakeholder-centric governance frameworks moderate the salience of financial incentives relative to reputational or relational capital.

While our analysis centers on CEO motivation and compensation structures, future research should account for external governance mechanisms, particularly the growing role of institutional investors and activist shareholders in shaping executive incentives. In contexts like Germany and Scandinavia, blockholders and long-term funds actively engage with boards on compensation and risk strategy [214]. Poland, though still evolving, has seen increased involvement of pension and insurance funds, particularly in firms listed on the WSE. Such actors may constrain or amplify the motivational pathways explored in our model, signaling a need to consider ownership structures and governance activism in compensation-risk linkages. Such actors may selectively reinforce either caution or boldness, depending on their investment horizon, activism style, and engagement strategy.

While our hypotheses posit linear relationships, non-linear effects are also plausible. Related conceptual work suggests that executives’ responses to compensation arrangements may vary with individual characteristics [94]. Future models may benefit from testing for curvilinear associations and interaction thresholds. Moreover, future models may explore asymmetric curvilinear effects, where excessive bonuses may backfire for promotion-focused CEOs while remaining motivational for prevention-focused counterparts.

We acknowledge the possibility of reverse causality. Although our design uses lagged independent variables, where regulatory focus and compensation are measured in year t–1 to predict strategic risk in year t, it remains plausible that firms with certain strategic orientations may recruit or retain CEOs with corresponding motivational profiles (e.g., high-risk firms selecting promotion-focused leaders). While our longitudinal structure mitigates simultaneous causation [215], the lack of a valid instrumental variable (IV) prevents us from definitively ruling out endogeneity. Future studies may consider natural experiments or exogenous shocks to regulatory focus or governance mechanisms to address this issue. Quasi-experimental designs leveraging exogenous CEO turnover or regulatory shocks could also help establish causal inferences.

Our analysis focuses on compensation as a moderator of the regulatory focus–risk-taking relationship. However, this relationship may also be contingent on firm-specific attributes, such as size, age, or board vigilance. For instance, larger firms may impose more governance constraints on CEO behavior [68,170]. Interaction effects with firm governance characteristics or external monitoring institutions may offer a more complete picture of when compensation mechanisms are effective. Future work could test whether board independence, CEO duality, or stakeholder salience further moderate these effects.

Moreover, our hypotheses were framed as unidirectional, assuming consistent effects of regulatory focus and compensation across contexts. Yet, in periods of economic crisis or regulatory tightening, risk behavior may deviate from these patterns. Prior studies show that situational stressors or stakeholder pressures can override personal dispositions [162,165]. Future research could examine whether contextual volatility moderates or reverses the direction of hypothesized effects. In particular, economic shocks or intense stakeholder scrutiny may suppress natural dispositional tendencies, producing behavior inconsistent with baseline expectations and highlighting the need for more context-sensitive models of executive decision-making.

## Conclusions

The primary aim of this study was to examine the heterogeneity of CEOs’ risk preferences and how they are shaped by fixed and variable compensation components implemented as part of corporate governance policies. This was achieved through theoretical, methodological, and practical dimensions.

On the theoretical level, we identified relevant frameworks, emphasizing risk at both the managerial and organizational levels. The literature review highlighted diverse approaches to defining and measuring risk, revealing a dual focus: strategic risk viewed at the manager or firm level. The analysis of the literature formed the basis for defining strategic managerial risk as firm-level strategic decisions reflecting CEOs’ choices. This definition underscores the distinction between firm and managerial interests and the importance of aligning external incentives with individual CEO traits.

Reviewing theories on top executives’ risk-taking revealed that separating individual and situational factors is complex. We aimed for an integrated model, synthesizing perspectives from management (UET, BAM, AT) and psychology (PT, RFT), while also accounting for institutional contexts. In particular, we analyzed how global corporate governance models shape executive incentives, noting that non-U.S. systems tend to emphasize long-term growth over short-term stock performance. This shift may help explain the less frequent use of high-powered, risk-increasing incentives.

Building on this foundation, our empirical findings support the notion that promotion- and prevention-focused orientations are stable motivational traits that significantly predict firm-level strategies. Moreover, these psychological dispositions interact with compensation mechanisms: both fixed and variable pay components moderate how motivation translates into risk-related behavior. This suggests that the same incentive may produce divergent outcomes depending on the executive’s regulatory focus – an important extension of the BAM and a step toward a more context-sensitive theory of behavioral governance.

Methodologically, our main contribution lies in the development and implementation of a novel measurement approach for CEO regulatory focus in a European context, particularly in Poland, and evaluating its influence on strategic risk. This was achieved using a validated Polish version of the LIWC dictionary to analyze CEOs’ shareholder letters, developed in collaboration with linguistic and psychological experts and published in [147]. Our approach enabled objective identification of promotion or prevention foci based on authentic textual expressions, offering a viable alternative to survey-based or experimental methods, which are often limited in large-scale studies.

Although our sample included only 82 firms from WSE, this limitation reflects broader challenges facing researchers in low-transparency markets such as Poland, where executive compensation data became systematically available only after 2020. This highlights the need for improved data access in European contexts and the dominant role of U.S.-based datasets in the literature. Nonetheless, the robustness of our findings is reinforced by a multi-step methodology, including sample selection corrections using the Heckman two-stage model and robustness checks based on alternative proxies for strategic risk (e.g., M&A activity, foreign expansion).

Empirical findings confirmed that regulatory focus significantly impacts CEOs’ strategic decisions. Both promotion and prevention focus influenced risk-taking, reflected in R&D spending, CAPEX, and long-term debt. Promotion-focused leaders were more likely to undertake bold investments, while prevention-focused CEOs adopted more conservative financial decisions. These baseline patterns align with the logic of RFT.

Compensation moderated these effects in nuanced ways. Bonuses increased risk-taking among prevention-focused CEOs (consistent with our hypotheses) but exacerbated risk-taking in promotion-focused CEOs, contrary to agency-theoretic expectations. Fixed salaries had a general dampening effect on risk-taking across the board but were especially effective for prevention-oriented executives. For promotion-focused CEOs, high base pay somewhat reduced their aggressiveness, suggesting that even inherently bold leaders respond to financial stability by reducing dynamism.

These findings have several practical implications. Boards should integrate motivational profiling into executive selection and performance evaluation. Knowing whether a CEO is naturally risk-seeking or risk-averse should guide compensation design. For instance, promotion-focused executives may require stabilizing incentives, such as greater fixed pay, capped bonuses, or deferred equity components with clawback provisions. These tools may help limit excessive risk-taking fueled by overconfidence or short-termism. Conversely, prevention-focused CEOs may benefit from well-targeted bonuses tied to specific strategic outcomes, such as entry into new markets or implementation of innovation initiatives, that gently encourage calculated risk-taking without compromising the firm’s overall stability. Such incentive architectures should “nudge” conservative executives outside their comfort zones while maintaining accountability.

Furthermore, prior comparative research has documented cross-national differences in executive compensation and corporate governance [216]. The implications of our findings therefore extend beyond individual firms to governance systems across countries. In shareholder-centric models (e.g., U.S.), where performance-linked pay is widespread, our findings support the introduction of ex-ante safeguards such as malus/clawback provisions, bonus caps, or long-term vesting conditions to prevent excessive behavior among promotion-driven CEOs. In stakeholder-oriented models (e.g., Germany, Scandinavia), where stability and consensus are prioritized [32], incentives for prevention-oriented CEOs can be used more assertively, as broader governance mechanisms act as risk-mitigating buffers. In hybrid models such as those in Central and Eastern Europe, which combine elements of insider- and outsider-oriented governance traditions [111], a balanced approach is needed: combining moderate incentive intensity to promote growth with appropriate controls to prevent abuse. In such an environment, incentives should be complemented by safeguards (e.g., long-term KPIs, ESG metrics, or peer-based benchmarking) to align risk-taking with broader stakeholder expectations. Risk calibration should be sensitive not only to market conditions but also to executives’ dispositional orientations. Instruments such as balanced scorecards or deferred bonuses tied to long-term sustainability metrics may help mitigate unintended risk amplification among promotion-focused CEOs.

We also recommend that board-level expertise in behavioral governance be enhanced, particularly through training in decision psychology and incentive design. Understanding that the same incentive does not work equally for all executives should become part of supervisory culture. Such a shift would help prevent mismatches between executive style and firm needs, whether by avoiding the hiring of overly cautious leaders in dynamic markets or overly aggressive leaders in sensitive contexts.

Finally, regulatory implications should not be overlooked. Transparency reforms like SRD II have improved public oversight of remuneration, but further steps are possible. For instance, companies could be encouraged (or required) to include a behavioral rationale in their remuneration reports, explaining how a given compensation structure supports strategic goals given the CEO’s known leadership profile. This would promote stakeholder trust and encourage boards to consciously manage behavioral risk.

In conclusion, this study reinforces the relevance of RFT to corporate strategic management and deepens our understanding of how the structure of executive incentives may produce differentiated outcomes depending on who receives them. We challenge the linear, universalist view of incentive–behavior relationships, emphasizing instead that the interaction between motivation and incentive design is both theoretically rich and practically consequential. This supports a broader movement toward personalized, psychology-informed governance strategies in today’s complex organizational environments.

## Supporting information

S1 Table Sectoral Distribution of Firms Included in the Final Analytic Sample (N = 82). (DOCX)

S2 Table Probit Model Estimating the Probability of Inclusion in the Final Analytic Sample (Used in Heckman Correction for Selection Bias in Fixed-Effects Models). (DOCX)

S3 Table Probit Model Estimating the Probability of CEO Succession (Used in Heckman Correction for Succession Bias in Fixed-Effects Models). (DOCX)

S4 Table Modeling Managerial Strategic Risk: Fixed-Effects versus Random-Effects Estimates with Heckman Selection Adjustment. (DOCX)

S1 Fig Conceptual Model of Strategic Managerial Risk. (DOCX)

S2 Fig CEO Promotion Focus and Bonus. (DOCX)

S3 Fig CEO Prevention Focus and Bonus. (DOCX)

S4 Fig CEO Promotion Focus and Fixed Compensation. (DOCX)

S5 Fig CEO Prevention Focus and Fixed Compensation. (DOCX)
