# Supplementary material for: Promote or prevent? A regulatory focus perspective on managerial risk taking
Source: PLoS One. 2026 Jul 31;21(7):e0352905. doi: 10.1371/journal.pone.0352905 (PMC13426988; doi:10.1371/journal.pone.0352905)
Supplement: S1 Table — (DOCX) [file pone.0352905.s001.docx]

**S1 Table. Sectoral Distribution of Firms Included in the Final Analytic Sample (N = 82).**

| **Sector Code** | **Sector** | **Number of firms** | **Percent** |
| --- | --- | --- | --- |
| 2 | Construction and Real Estate | 18 | 21.95 |
| 4 | Finance and Insurance | 17 | 20.73 |
| 6 | Manufacturing – Heavy Industry | 9 | 10.98 |
| 8 | Media and Communication | 9 | 10.98 |
| 7 | Manufacturing – Light and Consumer Goods | 8 | 9.76 |
| 5 | Health and Services / IT and Technology | 7 | 8.54 |
| 1 | Agriculture and Food | 5 | 6.1 |
| 3 | Energy and Utilities | 4 | 4.88 |
| 9 | Residual category: Miscellaneous and Unclassified | 2 | 2.44 |
| 10 | Trade and Retail | 2 | 2.44 |
| 11 | Transport and Logistics | 1 | 1.22 |
|  | Total | 82 | 100 |

***Source:****Own calculations based on classification of firms by primary industry. Sector codes correspond to internal identifiers used during sample construction.*
