# Supplementary material for: Promote or prevent? A regulatory focus perspective on managerial risk taking
Source: PLoS One. 2026 Jul 31;21(7):e0352905. doi: 10.1371/journal.pone.0352905 (PMC13426988; doi:10.1371/journal.pone.0352905)
Supplement: S2 Table — (DOCX) [file pone.0352905.s002.docx]

**S2 Table. Probit Model Estimating the Probability of Inclusion in the Final Analytic Sample (Used in Heckman Correction for Selection Bias in Fixed-Effects Models).**

This first-stage probit model estimates the likelihood that a firm-year observation is included in the final analytic sample (i.e., firms with complete CEO compensation and shareholder data). Predictors include firm-level characteristics unlikely to directly influence strategic risk-taking: firm size (lnRevenue), profitability (ROA), CEO ownership, and sector. The estimated Inverse Mills Ratio (IMR) from this model is included in the main fixed-effects regressions to adjust for non-random sample selection.

| **Variable** | **Coefficient** | **Std. Err.** | **z** | **P>\|z\|** | **[95% Conf. Interval]** | |
| --- | --- | --- | --- | --- | --- | --- |
| ROA | -0.00003 | 0.00007 | -0.42 | 0.671 | -0.00017 | 0.00011 |
| lnRevenue | 0.21610 | 0.01341 | 16.12 | 0.000 | 0.18982 | 0.24238 |
| CEO ownership | -0.22212 | 0.15549 | -1.43 | 0.153 | -0.52688 | 0.08264 |
| Sector = 2 | 0.33077 | 0.12110 | 2.73 | 0.006 | 0.09342 | 0.56812 |
| Sector = 3 | -0.00962 | 0.16337 | -0.06 | 0.953 | -0.32981 | 0.31057 |
| Sector = 4 | 0.68979 | 0.12810 | 5.38 | 0.000 | 0.43871 | 0.94087 |
| Sector = 5 | 0.07195 | 0.13336 | 0.54 | 0.590 | -0.18943 | 0.33334 |
| Sector = 6 | 0.01838 | 0.13296 | 0.14 | 0.890 | -0.24222 | 0.27897 |
| Sector = 7 | -0.15242 | 0.12967 | -1.18 | 0.240 | -0.40656 | 0.10172 |
| Sector = 8 | 0.77751 | 0.14179 | 5.48 | 0.000 | 0.49962 | 1.05540 |
| Sector = 9 | -0.03282 | 0.18247 | -0.18 | 0.857 | -0.39046 | 0.32482 |
| Sector = 10 | 0.37978 | 0.19556 | 1.94 | 0.052 | -0.00351 | 0.76307 |
| Sector = 11 | -0.43070 | 0.22875 | -1.88 | 0.060 | -0.87904 | 0.01765 |
| Constant | -3.49605 | 0.21349 | -16.38 | 0.000 | -3.91449 | -3.07762 |

Note: Sector dummies are included for Sectors 2–11; Sector 1 serves as the omitted reference group.

**Model statistics:**

Number of observations: Number of observations: 2,895 firm-year observations from 310 unique firms listed on the Warsaw Stock Exchange.

LR χ²(13): 408.27

Pseudo R²: 0.1190

Log likelihood: –1511.90

Unit of analysis: Firm-year (from 310 firms on the Warsaw Stock Exchange)

**Supplementary Note: Sector Classification**

Sector classification is based on aggregation of 82 original Notoria industry categories into 11 macro-sectors to increase model stability and interpretability. Sector 9 combines firms that did not clearly fit any of the other defined groups.

| Sector Code | Aggregated Sector Name |
| --- | --- |
| 1 | Agriculture and Food |
| 2 | Construction and Real Estate |
| 3 | Energy and Utilities |
| 4 | Finance and Insurance |
| 5 | Health and Services / IT and Technology |
| 6 | Manufacturing – Heavy Industry |
| 7 | Manufacturing – Light and Consumer Goods |
| 8 | Media and Communication |
| 9 | Residual category: Miscellaneous and Unclassified |
| 10 | Trade and Retail |
| 11 | Transport and Logistics |
