# Supplementary material for: Promote or prevent? A regulatory focus perspective on managerial risk taking
Source: PLoS One. 2026 Jul 31;21(7):e0352905. doi: 10.1371/journal.pone.0352905 (PMC13426988; doi:10.1371/journal.pone.0352905)
Supplement: S3 Table — (DOCX) [file pone.0352905.s003.docx]

**S3 Table. Probit Model Estimating the Probability of CEO Succession (Used in Heckman Correction for Succession Bias in Fixed-Effects Models).**

| Succession | Coefficient | Std. err. | z | P>\|z\| | [95% conf. interval] | |
| --- | --- | --- | --- | --- | --- | --- |
| Revenue (ln) | 0,234 | 0,051 | 4,610 | 0,000 | 0,134 | 0,334 |
| ROA | -2,305 | 0,459 | -5,030 | 0,000 | -3,204 | -1,407 |
| CEO ownership | -0,435 | 0,057 | -7,570 | 0,000 | -0,547 | -0,322 |
| Constant | 0,130 | 0,050 | 2,610 | 0,009 | 0,032 | 0,228 |

Number of obs* = 820

LR chi2(3) = 153.85

Prob > chi2 = 0.0000

Pseudo R2 = 0.1314

Log likelihood = -508.5322

*Unit of analysis: firm-year (N = 820 observations from 82 firms)
