# Supplementary material for: Promote or prevent? A regulatory focus perspective on managerial risk taking
Source: PLoS One. 2026 Jul 31;21(7):e0352905. doi: 10.1371/journal.pone.0352905 (PMC13426988; doi:10.1371/journal.pone.0352905)
Supplement: S4 Table — (DOCX) [file pone.0352905.s004.docx]

S4 Table. Modeling Managerial Strategic Risk: Fixed-Effects versus Random-Effects Estimates with Heckman Selection Adjustment.

| Variables | Model FE | | Model RE | |
| --- | --- | --- | --- | --- |
|  | Coefficient (Std. Err.) | p-value | Coefficient (Std. Err.) | *p*-value |
| Promotion focus | 0.570*** (0.080) | 0.0 | 0.455*** (0.080) | 0.0 |
| Prevention focus | -0.242* (0.12) | 0.041 | -0.977*** (0.157) | 0.0 |
| Promotion focus x CEO bonus | 0.275* (0.108) | 0.042 | 0.239 (0.164) | 0.144 |
| Prevention focus x CEO bonus | 0.152* (0.062) | 0.031 | 0.083 (0.095) | 0.383 |
| Promotion focus x CEO fixed compensation | -0.190** (0.076) | 0.013 | -0.186** (0.078) | 0.017 |
| Prevention focus x CEO fixed compensation | -0.168** (0.068) | 0.016 | -0.192 (0.070) | 0.193 |
| CEO fixed compensation (ln) | -2.445*** (0.173) | 0.0 | -1.925*** (0.161) | 0.0 |
| CEO bonus (ln) | 1.132*** (0.174) | 0.0 | 0.903*** (0.173) | 0.0 |
| ROA | -1.386*** (0.512) | 0.007 | -1.286** (0.533) | 0.016 |
| CEO ownership | -0.077 (0.122) | 0.529 | 0.244** (0.095) | 0.01 |
| CEO tenure | -0.154* (0.085) | 0.07 | -0.182** (0.082) | 0.027 |
| Revenues (ln) | -1.916** (0.765) | 0.013 | -3.915*** (0.454) | 0.0 |
| CEO gender |  |  | 0.579** (0.251) | 0.021 |
| Sector |  |  | 0.030 (0.550) | 0.576 |
| Succession |  |  | 0.308** (0.100) | 0.022 |
| IMR sample | 13.297*** (0.217) | 0.0 | 0.316 (0.607) | 0.603 |
| IMR succession | -16.808*** (0.979) | 0.0 | -13.346*** (0.823) | 0.0 |
| y_2012 | -0.668*** (0.164) | 0.0 | -0.639*** (0.169) | 0.0 |
| y_2013 | -0.671*** (0.160) | 0.0 | -0.632*** (0.166) | 0.0 |
| y_2014 | -0.661*** (0.157) | 0.0 | -0.624*** (0.163) | 0.0 |
| y_2015 | -0.508*** (0.157) | 0.001 | -0.471*** (0.163) | 0.004 |
| y_2016 | -0.607*** (0.158) | 0.0 | -0.554*** (0.164) | 0.001 |
| y_2017 | -0.465*** (0.158) | 0.003 | -0.426*** (0.164) | 0.009 |
| y_2018 | -0.451*** (0.156) | 0.004 | -0.477*** (0.162) | 0.003 |
| y_2019 | -0.318* (0.164) | 0.054 | -0.412** (0.171) | 0.016 |
| y_2020 | 0.189 (0.185) | 0.309 | 0.122 (0.192) | 0.525 |
| Constant | 15.786*** (1.978) | 0.0 | 25.553*** (0.826) | 0.0 |
| Observations | 573 |  | 572 |  |
| Groups | 80 |  | 80 |  |
| R-squared (within) | 0.560 |  | 0.541 |  |
| R-squared (between) | 0.186 |  | 0.784 |  |
| R-squared (overall) | 0.126 |  | 0.751 |  |

***Note****: Standard errors in parentheses.*

**** p < 0.001, ** p < 0.01, * p < 0.05.
Year 2011 is the reference category and omitted from the models. Year dummies (2012–2020) are included in both models unless excluded due to collinearity.*

*Time-invariant control variables, such as CEO gender and CEO succession, were excluded from the fixed-effects models due to collinearity or lack of within-firm variation. Sample- and succession-related selection biases were addressed using the Inverse Mills Ratio (IMR) derived from two-step Heckman correction models.*

*Source: Own calculations.*
