# Supplementary material for: Promote or prevent? A regulatory focus perspective on managerial risk taking
Source: PLoS One. 2026 Jul 31;21(7):e0352905. doi: 10.1371/journal.pone.0352905 (PMC13426988; doi:10.1371/journal.pone.0352905)
Supplement: S1 Fig — (DOCX) [file pone.0352905.s005.docx]

S1 Fig. Conceptual Model of Strategic Managerial Risk.

H1 (+)

**Promotion focus**

**Prevention focus**

**Strategic managerial risk**

**CEO bonus**

**CEO fixed compensation**

H4 (+)

H6 (-)

H3 (-)

H5 (+)

H2 (-)

Source: own work.
