# Supplementary material for: Promote or prevent? A regulatory focus perspective on managerial risk taking
Source: PLoS One. 2026 Jul 31;21(7):e0352905. doi: 10.1371/journal.pone.0352905 (PMC13426988; doi:10.1371/journal.pone.0352905)
Supplement: S5 Fig — (DOCX) [file pone.0352905.s009.docx]

S5 Fig. CEO Prevention Focus and Fixed Compensation.

Note: High and low prevention focus (x-axis) represent one standard deviation above and below the mean, respectively. Similarly, high and low fixed compensation reflect one standard deviation above and below the mean.

Source: own work.
