# Supplementary material for: Promote or prevent? A regulatory focus perspective on managerial risk taking
Source: PLoS One. 2026 Jul 31;21(7):e0352905. doi: 10.1371/journal.pone.0352905 (PMC13426988; doi:10.1371/journal.pone.0352905)
Supplement: Attachment — Submitted filename: Response to Reviewers.docx [file pone.0352905.s012.docx]

**Response to Reviewer Comments**

**on the Manuscript Entitled *Promote or Prevent? A Regulatory Focus Perspective on Managerial Risk Taking***

We sincerely thank the reviewers for their detailed and constructive feedback. We have carefully addressed each comment and substantially revised the manuscript in line with the concerns raised. These revisions have resulted in significant improvements in the theoretical positioning, methodological transparency, and interpretive depth of our study.

Most importantly, in response to the methodological concerns regarding sample composition, we excluded seven firms listed on non-Polish stock exchanges. This adjustment led to updated empirical models and the recalculation of all regression results. Consequently, Tables 1–3, Supplementary Figures S2–S5, and Supplementary Tables S1–S4 have been revised accordingly. While these revisions slightly modified specific coefficient estimates, they did not alter the overall direction or substantive interpretation of our findings. The core theoretical claims and empirical conclusions remain robust.

We have also strengthened the integration of theory and results, enhanced the clarity and specificity of the practical implications, and expanded the discussion of limitations and future research directions. Several new sections were added (e.g., in the *Method*, *Results*, and *Discussion*), and others were significantly restructured to reflect a more critical engagement with the data and prior literature.

Below, we respond point-by-point to each reviewer comment. For each issue raised, we summarize our revisions and indicate where in the manuscript the relevant changes have been made (with page and line references, where applicable). We trust that these revisions adequately address the reviewer’s concerns and improve the quality and coherence of the manuscript.

**Reviewer #1:** *(1.) Lack of Clear Research Gap and Theoretical Contribution. (1.1.) While the study discusses the role of regulatory focus in managerial decision-making, it does not sufficiently articulate its novelty relative to prior literature. The connection between CEO compensation and risk preferences has been widely studied within agency theory and behavioral finance, yet the paper does not explicitly delineate how its findings extend or challenge existing paradigms.*

**Reviewer #2:** *(1.) I believe that the theoretical foundation could better explain how and why different compensation structures impact risk-taking, perhaps with a clearer link between Regulatory Focus Theory and Behavioral Agency Theory.*

**Response:**
We thank both reviewers for highlighting the need to better articulate the contribution of our study. We have revised the manuscript to emphasize the novelty of integrating RFT – a psychological framework – into the corporate governance literature, particularly in connection with compensation design. While previous studies (e.g., Sanders & Hambrick, 2007; Beatty & Zajac, 1994) examined the link between executive pay and risk-taking, few have explored how dispositional motivation moderates this relationship.

We now make clear that our study addresses this gap by demonstrating how RFT can explain behavioral heterogeneity in CEO responses to identical incentive structures. This approach advances existing theories (AT, BAM, UET) by accounting for individual-level variation in motivational orientation, a dimension often overlooked in governance research (**pp. 5-6, lines 110–136**).

**Reviewer #1:** *(1.2.) The introduction lacks a critical synthesis of past studies and fails to clearly differentiate this study from prior empirical investigations.*

**Response:**
We thank the reviewer for this insightful observation. In response, we have revised the *Introduction* section to provide a more explicit and critical synthesis of prior literature and to highlight how our study extends existing research in meaningful ways.

Specifically, we added a new integrative paragraph (**see p. 20, lines 462–468**) that outlines the empirical limitations of previous studies focusing on CEO risk-taking. We emphasize that prior research has largely relied on U.S.-centric data and focused either on structural variables (e.g., incentive alignment, power asymmetries) or dispositional traits in isolation, without systematically integrating motivational orientation (as conceptualized by Regulatory Focus Theory) with corporate governance mechanisms. Furthermore, we note that even when RFT is included in earlier studies (e.g., Gamache et al., 2015; Lanaj et al., 2012), it is typically treated as a secondary moderator rather than a core explanatory variable.

By embedding RFT within a broader theoretical framework that includes BAM, PT, and UET, and by testing this integration empirically in a hybrid governance environment (Poland), our study provides a cross-contextual, psychologically informed explanation of CEO strategic risk-taking. This approach goes beyond prior research by demonstrating how the same formal incentives may elicit divergent behaviors depending on stable motivational orientation. Thus, we now clearly position our contribution as a theoretically novel and empirically grounded advancement in the behavioral governance literature.

We hope these revisions directly address the reviewer’s concern and clarify how our study builds upon and differentiates itself from earlier empirical investigations.

**Reviewer #1:** *(1.3.) Although regulatory focus theory (RFT) is well-established in psychology and management research, the article does not integrate it with competing governance perspectives, such as the Upper Echelons Theory (UET) or Prospect Theory, which also address CEO decision-making biases. For instance, Sewpersadh (2019) examines different types of CEO power (structural, expert, prestige) and how these influence strategic decision-making. This provides a critical lens for evaluating how CEO compensation aligns with different power structures. Sewpersadh, N.S. (2019), "An examination of CEO power with board vigilance as a catalyst for firm growth in South Africa", Measuring Business Excellence, Vol. 23 No. 4, pp. 377-395. https://doi.org/10.1108/MBE-10-2018-0083*

**Response:**
We appreciate the reviewer’s suggestion to more clearly situate Regulatory Focus Theory (RFT) within the broader landscape of executive decision-making frameworks. In response, we have revised the theoretical section to integrate RFT with both Upper Echelons Theory (UET) and Prospect Theory (PT). Specifically, we now clarify that RFT captures stable, chronic motivational orientations (promotion vs. prevention focus), whereas UET emphasizes observable executive traits and PT focuses on situational framing of decisions. By aligning RFT with these established frameworks, we offer a complementary psychological lens that enriches our understanding of CEO-level variability in risk preferences under identical structural conditions (**pp. 3–4, lines 63–88**).

Additionally, we explicitly contrast our dispositional approach with power-based governance models such as Sewpersadh (2019), emphasizing that our contribution shifts the analytical focus from positional authority to internal motivational states. We believe this integration addresses the reviewer’s concern and strengthens the theoretical coherence of the study.

**Reviewer #1:** *(1.4.) The study does not explore counterarguments or limitations of applying RFT in the CEO compensation context, leaving theoretical gaps unaddressed.*

**Response:**

In the revised *Discussion*, we explicitly address potential limitations of applying RFT to CEO behavior. We note that while RFT offers a robust dispositional lens, it does not account for all situational constraints imposed by organizational governance or external monitoring. For instance, the expected moderating effects of compensation on promotion-focused CEOs were not supported, suggesting that financial incentives may be less influential in shaping risk behavior for this group. We now interpret this as a possible boundary condition for the applicability of RFT in high-status executive roles, where agency discretion may be tempered by power dynamics or pre-existing strategic mandates (**see pp. 49–50, lines 1044–1053** in *Discussion*). We have also signaled the need for future research to test whether RFT effects hold under varying institutional logics and leadership contexts.

**Reviewer #1:** *(2.) Methodological Concerns. The empirical design, while employing longitudinal panel data, has several shortcomings that undermine the robustness and generalizability of the findings. (2.1) Sampling and external validity. (2.1.1.) The study relies on a relatively small sample of 89 firms, predominantly from the Warsaw Stock Exchange (WSE), limiting its applicability to broader corporate governance contexts.*

**Response:**
In the revised manuscript, we explicitly acknowledge this limitation (**see p. 52, lines 1019–1020**, section: *Limitations and future research directions*), clarifying that the final sample of 82 firms (resulting from the exclusion of 7 companies listed on non-Polish stock exchanges – see reviewer comment below) reflects a data-driven sampling logic (see also **p. 21, lines 497–506**, and **p. 22, lines 509–511**). This logic prioritizes the availability of complete and high-quality data (CEO letters, compensation, and strategic indicators) over full population coverage. Such a sampling approach is common in management and behavioral research, where disclosure practices vary significantly across jurisdictions.

In Poland, there is no strict obligation to disclose many types of corporate information beyond core financial statements for publicly listed companies. However, recent legal and market developments indicate a clear trend towards greater transparency. Limited access to data that is widely available in other jurisdictions, such as the United States, poses a serious challenge for researchers in many countries, including those in Central and Eastern Europe. This affects the size of usable, high-quality samples and represents both a limitation and a structural research challenge for scholars working in these contexts.

To mitigate potential sample selection bias arising from non-random availability of CEO letters and compensation data – which may be correlated with unobserved determinants of strategic risk – we applied the two-step Heckman correction procedure (Heckman, 1979). This procedure, described in detail later in our response, allowed us to generate the inverse Mills ratio (IMR), which was included in both the fixed effects (FE) and random effects (RE) models (see Method section).

Although the primary empirical focus is on Poland, the findings can be analytically extended to other continental European governance systems that share similar institutional characteristics and a hybrid corporate model (**p. 56, lines 1194–1203**, section: *Limitations and future research directions*). The study contributes to the growing body of research on executive behavior outside the United States, particularly within the underrepresented context of Central and Eastern Europe.

**Reviewer #1:** *(2.1.2.) The inclusion of a few firms from other European exchanges (e.g., Germany, Spain, Portugal) is inconsistent and lacks a clear justification.*

**Response:**
In the revised version of the manuscript (see **p. 21, lines 497–506**, and **p. 22, lines 509–511**), we decided to exclude the 7 companies listed outside the Polish stock exchange. Their initial inclusion was motivated by the availability of comparable longitudinal data on CEO communication and compensation, given the significant data gaps observed among companies listed on the Warsaw Stock Exchange (WSE), particularly regarding remuneration, bonuses, and CEO letters to shareholders. However, upon further reflection, we fully agree with the reviewers that such a mixed sample lacks consistency and that the justification for its inclusion was insufficient.

**Reviewer #1:** *(2.1.3.) There is no discussion of industry-specific risk factors that might influence CEO decision-making differently across sectors.*

**Reviewer #2:** *(5.) Fixed salaries appear to reduce risk-taking in promotion-focused CEOs. I wonder if the authors could discuss a little bit governance or industry constraints that could explain this result.*

**Response:**
In the revised version of the manuscript, we have included a sector control variable and indicated the distribution of companies in the sample across 11 sectors (as detailed in the sample description on **p. 22, lines 512–521** and in *Supplementary Table S1*). Unfortunately, this variable was automatically excluded from the main fixed effects (FE) models due to its time-invariant nature, which is a known limitation of FE estimation. However, it was included in the robustness check using a random effects (RE) model, where it did not display a statistically significant relationship with the dependent variable – managerial strategic risk.

This does not imply that sectoral context is irrelevant in our research. We fully acknowledge that limitations of the sample prevented a proper examination of sectoral conditioning, and our model does not fully capture sector-specific risk norms. We recognize this as a limitation and emphasize the need for more in-depth treatment of this variable in future studies. This limitation is illustrated by contrasting highly regulated sectors (e.g., banking) with less regulated ones (e.g., technology). We also highlight that sectoral conventions are likely to shape how CEOs perceive strategic boldness – either as innovation or recklessness – depending on the industry context. This insight provides a foundation for future research involving sector-specific sampling or interaction terms (see **pp. 53–54, lines 1134–1148** in the section *Limitations and future research directions*).

**Reviewer #1:** *(2.1.4.) The omission of small and medium-sized enterprises (SMEs) creates a selection bias, as SMEs often face different governance and risk dynamics compared to publicly traded firms.*

**Response:**
We acknowledge this limitation and have explicitly addressed it in the revised manuscript. In the updated section, we now clarify that the sample includes only publicly listed firms and discuss how this introduces selection bias, as SMEs may exhibit distinct governance mechanisms and risk dynamics (Berger & Udell, 2006). We also highlight that CEO ownership is typically higher in SMEs, which may amplify or dampen the effects of regulatory focus. We encourage future studies to examine whether our findings replicate in SME contexts, where incentive structures are often less formalized and leadership effects more personalized (see **p. 53, lines 1125–1133** of *Limitations and future research directions*).

**Reviewer #1:** *(2.2) Measurement Limitations. CEO regulatory focus is inferred through content analysis of shareholder letters using the Polish LIWC dictionary. However, the study does not critically engage with potential biases in this methodology. (2.2.1.) The assumption that shareholder letters authentically reflect CEO decision-making is problematic, as such letters may be influenced by public relations teams or investor relations advisors.*

**Reviewer #2:** *(2.) The use of shareholder letters to assess CEO regulatory focus is interesting, but I believe that some potential biases could be addressed. I wonder to what extent do these letters genuinely reflect CEO traits rather than corporate messaging strategies?*

**Response:**
We appreciate the reviewers’ concerns regarding the potential limitations of using shareholder letters to infer CEOs’ regulatory focus. In response, we have added a more extensive description of the content analysis methodology in the section detailing the measurement of our key psychological variables, including a justification for the choice of this method (**p. 23, lines 538–545**), and we explicitly address the possible influence of investor relations or public relations teams on the texts. We acknowledge that these publicly disclosed documents may not fully reflect unfiltered cognitive states. However, we clarify that our analysis aims to capture the externally communicated motivational emphasis of CEOs, rather than introspective content (see also the section *Limitations and future research directions*, **p. 54, lines 1149–1162**).

To strengthen the validity of our approach, we highlight that the Polish LIWC-based dictionary of regulatory orientation used in this study has been empirically validated in multiple studies, demonstrating content, discriminant, and correlational validity (Marszałek et al., 2023). This makes it a psychometrically robust tool for detecting promotion and prevention focus in Polish-language texts.

We also reinforce our methodological rationale by drawing on prior research (e.g., Gamache et al., 2015; Zheng et al., 2020), which successfully employed managerial discourse to infer dispositional traits. Moreover, we note that CEOs typically participate in drafting or thoroughly editing these letters and bear legal responsibility for their content, which supports the assumption that the language used reflects their strategic perspectives and motivational priorities.

In addition, we cite literature showing that linguistic style in corporate communication reflects stable psychological dispositions (Tausczik & Pennebaker, 2010), and that shareholder letters provide a discreet and consistent medium for long-term psychometric assessment (e.g., Craig & Amernic, 2011; Yekini et al., 2019). These characteristics offer practical advantages over self-report or survey-based methods, which often suffer from low response rates and social desirability bias.

We also suggest triangulating this method with interviews or behavioral data in future studies. Furthermore, we recommend exploring rhetorical or discursive models of sentiment analysis to capture deeper linguistic nuances (see **p. 54, lines 1149–1162** of the *Limitations and future research directions* section).

In sum, we believe that the revised manuscript critically addresses the assumptions and limitations of this method, while clearly articulating its empirical value and theoretical relevance for capturing CEOs’ motivational orientation.

**Reviewer #1:** *(2.2.2.) The linguistic sentiment analysis approach may fail to capture deeper, contextual nuances in CEO motivation, leading to measurement error.*

**Response:**
We appreciate the reviewer’s concerns regarding the limitations of linguistic sentiment analysis in capturing the full depth of CEOs’ motivational states. In response, we have expanded the methodological discussion to explicitly acknowledge this issue (see the *Variables* and *Limitations and future research directions* sections). In particular, we clarify that our use of LIWC-based content analysis should be understood as identifying publicly communicated motivations of CEOs, rather than serving as a direct and unfiltered indicator of internal cognitive processes (**p. 24, lines 560–562**).

While CEO letters are widely used in upper echelons and behavioral strategy research as a non-invasive and consistent source of data (e.g., Gamache et al., 2015; Zheng et al., 2020), we recognize that these documents may be partially shaped by investor relations professionals or communication teams. Accordingly, we interpret our findings as reflective of external strategic rhetoric, rather than internal psychological states per se.

To enhance the validity of our approach, we relied on a validated Polish regulatory focus dictionary (Marszałek et al., 2023), developed specifically for this linguistic and cultural context (see **p. 24, lines 570–576**). This tool has demonstrated both content and construct validity in experimental and correlational studies. We also emphasized the legal and reputational responsibility that CEOs bear when signing shareholder letters, which typically ensures their substantive involvement in the drafting process (e.g., Malhotra et al., 2018).

Finally, in the *Limitations and future research directions* section (**p. 54, lines 1149–1162**), we discuss the importance of triangulating linguistic measures with other data sources in future research – such as executive interviews, behavioral experiments, or longitudinal decision records – and we recommend exploring advanced rhetorical or discourse-based models to better capture the deeper contextual nuances of CEO communication.

**Reviewer #1:** *(2.2.3.) Compensation structures are treated simplistically. Annual bonuses and fixed salaries are analyzed, but the study does not account for long-term incentives, stock options, or equity-based compensation, which are common in executive pay structures.*

**Response:**
This limitation is now explicitly discussed in the manuscript. We acknowledge that equity-linked instruments play a significant role in CEO compensation in selected Polish firms (see **p. 15, lines 342–362**, and **p. 21, lines 497–506**). These include predominantly subscription warrants, but also stock options. Subscription warrants grant the right to acquire newly issued shares at a pre-determined price and are legally distinct from stock options, which typically confer rights to purchase existing shares and are often structured as contractual entitlements (Nowak, 2016; Gębusia, 2011). Despite their distinct legal classification, both instruments function similarly in aligning executive interests with shareholder value.

PwC (2021) reports that, in 2020, 44% of the long-term equity-based instruments used in variable compensation schemes among WSE-listed firms were subscription warrants. Importantly, our review of corporate remuneration disclosures indicates that realized equity-linked rewards are not always reported in a standalone “equity-based compensation” category, but are sometimes aggregated under annual bonuses. Consequently, while our bonus variable primarily captures short-term performance-based pay, in some cases it may partially reflect the value of exercised warrants or options. We recognize this as a limitation and a potential source of heterogeneity in bonus composition across firms (**pp. 54–55, lines 11****67–1175**).

**Reviewer #1:** *(2.2.4.) The classification of strategic risk-taking using R&D expenditures, long-term debt, and CAPEX is overly simplistic, as it does not distinguish between calculated strategic investments and reckless risk-taking.*

**Reviewer #2:** *(3.) The authors measure strategic risk primarily through financial indicators (R&D spending, CAPEX, long-term debt), but I wonder whether including qualitative aspects like strategic expansion decisions could enhance the analysis? Perhaps in authors’ future studies they could include alternative measures for strategic risk, like market expansion, innovation strategies etc.*

**Response:**
We fully acknowledge that the operationalization of strategic risk-taking through aggregated indicators such as R&D intensity, capital expenditures, and long-term debt does not allow us to differentiate between forward-looking, calculated strategies and ex post ineffective or reckless decisions. This limitation is inherent in most empirical research relying on financial proxies for latent constructs such as risk preferences or strategic boldness.

In response to the reviewers’ concern, we have clarified this important distinction in the manuscript. Specifically, we have added the following sentence to the paragraph describing the dependent variable:

“*While our strategic risk-taking variable reflects firm-level resource allocation under uncertainty – via R&D intensity, CAPEX, and long-term debt (Chng et al., 2012; Seo et al., 2015; Wallace et al., 2021) – we acknowledge this approach does not distinguish between visionary, calculated investments and imprudent risk-taking (March, 1991). For instance, high R&D intensity may signal innovation or reflect inefficient resource use. Similarly, leveraging via debt could indicate strategic expansion or excessive risk. Therefore, our measure should be interpreted as reflecting the intensity of strategic risk engagement rather than its normative quality or success. Importantly, our operationalization captures ex ante strategic commitments made under uncertainty, without assessing whether these decisions ultimately led to favorable”* (**pp. 26–27, lines 618–626**).

We believe this clarification makes explicit that the variable captures intended strategic engagement under risk rather than retrospective assessments of performance or judgment quality. We also refer the reviewer to the cited literature, which supports the validity of this measurement approach in top management and behavioral strategy research.

**Reviewer #1:** *(2.2.5.) There is no sensitivity analysis to test whether alternative measures of risk-taking (e.g., acquisitions, internationalization strategies) would yield consistent results.*

**Response:**
This issue is now directly addressed in the revised manuscript. We report a post-hoc robustness check using M&A activity and international expansion as proxy indicators of strategic risk-taking. Although data availability was limited, we found that firms engaged in M&A tended to score higher on our composite risk index, which lends support to the validity of our primary risk measure.

We also encourage future research to incorporate a broader range of strategic risk indicators – such as acquisition frequency or performance volatility – for more refined and context-sensitive operationalizations (**see p. 55, lines 1185–1193**, section Limitations and future research directions).

**Reviewer #1:** *(2.3.) Statistical Concerns. (2.3.1.) The study uses fixed-effects panel regression models, but there is no discussion of alternative specifications (e.g., random effects, instrumental variable approaches) to address potential endogeneity.*

**Response:**
We appreciate this insightful comment and have revised the manuscript to clarify the rationale for the model choice and to address concerns regarding endogeneity. In the newly added Method section (**pp. 30–31, lines 713-721**), we provide a more detailed justification for the use of fixed effects (FE) panel regression models. This decision is supported by a Hausman specification test, which indicated that the FE estimator was appropriate given the correlation between unobserved firm-level heterogeneity and the key predictors.

To assess the robustness of our findings, we additionally estimated random effects (RE) models including dummy variables that were automatically excluded from the FE models. These alternative specifications produced qualitatively similar results in terms of the direction, magnitude, and significance of the main explanatory variables (see *Supplementary Table S4*).

We also considered the use of instrumental variables (IV) or system GMM models to address potential simultaneity bias. However, the lack of strong and theoretically justified instruments in our dataset limited the feasibility of such approaches. Moreover, given our primary aim – analyzing within-firm changes in CEO behavior over time – the FE approach remains a conservative and appropriate estimation strategy, widely applied in strategic management and corporate governance research.

Nevertheless, we acknowledge that dynamic endogeneity cannot be entirely ruled out. We have added this as a limitation in the manuscript and identified it as a challenge for future research.

**Reviewer #1:** *(2.3.2.) The potential for reverse causality is not adequately controlled. It is plausible that firms with higher risk-taking tendencies select CEOs with a particular regulatory focus, rather than CEO traits solely driving firm behavior.*

**Response:**
We agree with the reviewer’s concern and have strengthened our discussion of potential endogeneity. Although our lagged research design helps to mitigate the risk of simultaneous causality, we acknowledge the limitation of not employing instrumental variables. In the revised manuscript, we now recommend the use of natural experiments, exogenous shocks, or CEO turnover events in future research to facilitate stronger causal inference (**see p. 57, lines 1224–1233**, section *Limitations and future research directions*).

**Reviewer #1:** *(2.3.3.) Multicollinearity is cited as a reason for excluding control variables (CEO gender, tenure), but no robustness checks are provided to assess how their exclusion affects results.*

**Response:**
In response, we have expanded the manuscript to clarify the rationale for excluding CEO gender and age in the fixed effects (FE) specifications, and we conducted additional robustness checks to assess the potential impact of omitting these variables.

As explained in the revised manuscript, CEO gender and age were initially considered as control variables due to their theoretical relevance. However, both variables were omitted from the fixed effects panel regressions due to their time-invariant or nearly constant nature within firms, which is a recognized limitation of FE models. In contrast, CEO tenure exhibited sufficient within-firm variation and was retained in all FE specifications. To assess multicollinearity, we calculated variance inflation factors (VIF) in the pooled OLS model and found that all values were below 3.2. Nevertheless, CEO gender was automatically dropped from the model due to collinearity, likely reflecting the very low proportion of female CEOs in the sample (<4%).

To directly address the reviewer’s request for robustness testing, we estimated random effects (RE) models and pooled OLS models with firm-level dummy variables, which allowed us to reintroduce CEO gender and age. These alternative model specifications confirmed that the inclusion of the previously excluded control variables did not materially alter the direction, magnitude, or statistical significance of the main effects. Specifically, CEO gender and age were not statistically significant in any of the models, and CEO tenure (which was retained throughout the analysis) showed only a marginal effect on strategic risk-taking. Full regression results from these robustness checks are presented in *Supplementary Table S4.*

We have incorporated this clarification into the manuscript to enhance transparency and analytical completeness (see **p. 30, lines 702–712**). We believe that these steps adequately address the reviewer’s concerns.

**Reviewer #1:** *(2.3.4.) The Heckman two-step correction for sample selection bias is mentioned but not fully detailed, leaving uncertainty about its effectiveness in mitigating bias.*

**Response:**
We thank the reviewer for pointing out the need for greater transparency regarding the implementation and effectiveness of the Heckman correction procedure. In response, we not only expanded the methodological explanation in the revised version of the manuscript (see the new Method section, **pp. 29–32, lines 690–763**), but also sought to enhance the rigor of our study by applying the Heckman procedure twice and generating two separate inverse Mills ratio (IMR) variables to address key limitations of our sample.

The first application addressed potential sample selection bias stemming from the non-random availability of CEO letters and compensation data, which may be correlated with unobserved determinants of strategic risk. To correct for this bias, we employed the two-step Heckman procedure (Heckman, 1979):

- In the first step, we estimated a probit model with random effects predicting inclusion in the final sample. The selection equation included predictors such as firm size, ROA, and CEO ownership status, which are associated with data availability but not theoretically linked to the outcome variable (i.e., managerial strategic risk). For this purpose, we used data from 310 WSE-listed firms for the period 2011–2020. The dependent variable in the probit model was a binary indicator denoting whether a firm was included (“1”) or not (“0”) in the target sample.
- The resulting IMR variable – named *IMR_sample* in the final dataset and models – was included as a covariate in the second-stage fixed effects regressions. The IMR coefficient was statistically significant (p < 0.001), indicating the presence of selection bias and the effectiveness of the correction.

For transparency, the full results of the selection model are presented in Supplementary Table S2. These additional details reinforce the robustness of our conclusions regarding the relationship between CEO regulatory focus, compensation, and strategic risk-taking. This procedure is described in detail on **p. 31, lines 729–740**.

The second application of the Heckman correction addressed the issue of CEO succession – i.e., the possibility that shareholder letters in a given firm over the 10-year period were authored by more than one CEO. In this case, a separate probit model was constructed with a binary dependent variable for succession, using similar predictors as in the previous model: firm size (lnRevenue), ROA, and CEO ownership (% of shares held). Full results of the selection model are presented in Supplementary Table S3, and the procedure is described on **p. 32, lines 743–758**.

The resulting IMR variable – named *IMR_succession* – was incorporated into the final models. As a result, our estimations include two correction terms which, in our view, enhance the methodological robustness of the study and directly address the reviewer’s concerns.

**Reviewer #1:** *(3.) Weaknesses in Hypothesis Development. (3.1.) The six hypotheses are overly deterministic, assuming linear relationships between regulatory focus, compensation, and risk-taking.*

**Reviewer #2:** *(4.) The finding that bonuses increase rather than temper risk-taking in promotion-focused CEOs challenges somehow the expectations. Is it possible the authors could give more details on alternative explanations, perhaps like intrinsic motivation or short-term financial goals?*

**Response:**
We recognize the value of investigating nonlinear dynamics and have addressed this issue in the revised version of the manuscript. We discuss the possibility of curvilinear and threshold effects – for example, the potential for very high bonuses to induce risk aversion or trigger reputational constraints. We cite prior research on inverted U-shaped relationships and encourage future studies to account for possible asymmetries or saturation effects in regulatory focus and compensation sensitivity (see **pp. 56–57, lines 1214–1223**, section *Limitations and future research directions*).

Importantly, our analyses – particularly those involving moderation effects – suggest the potential presence of nonlinear relationships, especially in the context of fixed compensation levels. Our findings may indirectly indicate that there exists a threshold level of fixed pay that alters the direction or intensity of the relationship between regulatory focus and managerial strategic risk-taking. This inverted U-shaped pattern thus emerges as a significant empirical challenge and a necessary avenue for future research.

**Reviewer #1:** *(3.2.) The study does not account for potential threshold effects, for instance, at what level of compensation does risk-taking behavior significantly change?*

**Response:**
While our current models treat compensation – both fixed and variable – as a continuous moderator, we acknowledge the possibility of nonlinear or threshold effects, whereby changes in CEO behavior may occur only after certain motivational levels are exceeded. In response, we have added a paragraph in the *Results* section (**p. 46, lines 971–981**), and a sentence in the *Discussion* section (**p. 49, lines 1038–1043**), recognizing that bonuses and salaries may moderate risk-taking behaviors only after surpassing a psychologically or symbolically meaningful level (Devers et al., 2008).

This addition clarifies the limitations of our linear specification and outlines a clear agenda for future research (see **p. 56–57, lines 1214–1223**, section *Limitations and future research directions*). Given the data constraints and sample size, we chose not to model thresholds empirically in the present study. However, we fully agree with the reviewer that investigating compensation thresholds would provide valuable insight into the motivational dynamics underlying strategic leadership behavior.

**Reviewer #1:** *(3.3.) The moderating effects of fixed and variable compensation are discussed, but no alternative models (e.g., interaction effects with firm size or governance structures) are considered.*

**Response:**
We have now addressed this gap. The revised section suggests that compensation effects may vary with firm-specific attributes such as size, age, or board independence. We recommend future work examining interaction effects with governance intensity or external monitoring mechanisms, which may condition when compensation structures are effective (see **p. 57, lines 1234–1240**, section *Limitations and future research directions*).

**Reviewer #1:** *(3.4.) Hypotheses are framed in unidirectional terms, without addressing conditions where relationships may not hold (e.g., economic downturns, regulatory changes).*

**Response:**
We have now added a discussion on situational moderators, noting that dispositional tendencies may be overridden by contextual stressors such as economic shocks or regulatory tightening. We cite prior work showing that stakeholder pressure and macro-level events can suppress trait-driven behavior and recommend further examination of such contingencies (see **p. 58, lines 1241–1249**, section *Limitations and future research directions*).

**Reviewer #1:** *(4.) Superficial Discussion of Findings. (4.1.) The discussion section restates statistical findings but lacks critical engagement with why certain hypotheses were not supported.*

**Reviewer #2:** *(2.3.) (7.) This is just a minor observation, about some statistical explanations in the results section are perhaps a little overly detailed and could be better placed in an appendix.*

**Response:**
In the revised version of the manuscript, we have significantly expanded and enriched the *Results* section (**pp. 32–47, lines 766–998**), including a strengthened interpretation of the findings – particularly those that were unexpected, such as the rejection of Hypotheses 3 and 5 (**see pp. 43–44, lines 894–912**). Rather than simply reporting, for example, that the interaction between promotion focus and bonuses was positive (contrary to expectations), we now provide a deeper theoretical explanation based on the concepts of regulatory fit (Bryan et al., 2011; Scholer et al., 2010) and strategic overconfidence among gain-oriented CEOs.

Similarly, regarding the unexpected moderating effect of fixed compensation, we now interpret the dampening effect of high fixed salaries – across both motivational profiles – as potentially reflecting loss aversion or a comfort zone effect, drawing on the literature on behavioral agency theory (Wiseman & Gomez-Mejia, 1998). These interpretations are clearly articulated in the concluding paragraphs on Model 5 and throughout the interaction analysis (**p. 42, lines 878–883**).

We acknowledge that these additions may somewhat conflict with Reviewer 2’s observation that the Results section may be overly detailed. Accordingly, we are open to revisiting the presentation of statistical details and moving more technical explanations to an appendix, should the reviewers feel that the level of detail in the Results section remains excessive.

**Reviewer #1:** *(4.2.) Unexpected findings, such as bonuses amplifying promotion-focused risk-taking rather than tempering it, are only briefly rationalized without deeper theoretical exploration.*

**Response:**
We agree with the reviewer that the initial explanation of this counterintuitive finding was too brief. We have now substantially deepened the theoretical rationale in the *Results* section. Specifically, we draw on regulatory focus theory to explain that promotion-focused individuals are particularly sensitive to gain-framed incentives and may experience amplified risk-taking under performance-contingent bonuses, rather than restraint. This behavior is interpreted through the lens of regulatory fit, where extrinsic rewards align with intrinsic motivational orientation, thus intensifying the behavioral response (Higgins, 1997; Bryan et al., 2011).

We also discuss the potential for overconfidence and reward salience effects, providing a more robust theoretical framing of the findings. This discussion has been placed adjacent to the presentation of Supplementary Figure S2, directly linking statistical results to theoretical interpretation (**p. 45, lines 947–954**).

**Reviewer #1:** *(4.3.) The implications for policymakers and corporate boards remain vague, with general recommendations rather than specific strategies for structuring CEO compensation in different governance environments.*

**Response:**
We appreciate this comment and have substantially revised the *Conclusions* section to provide more actionable and governance-specific recommendations. The revised text now articulates clear strategies tailored to different institutional contexts, including shareholder- and stakeholder-oriented regimes.

First, we emphasize that supervisory boards should integrate motivational profiling – in particular, CEOs’ regulatory focus – into their selection and compensation design processes. This suggestion is grounded in our empirical findings that motivational traits condition the behavioral effects of pay components.

Second, we now differentiate recommendations according to governance models. In shareholder-driven systems, performance-linked pay (e.g., bonuses) may be appropriate to stimulate bold strategic moves. However, in stakeholder-oriented contexts, such mechanisms may increase reputational risk or opportunistic behavior. To mitigate this, we recommend the use of balanced scorecards, deferred bonuses, or sustainability-linked metrics to align compensation with long-term value creation. This aligns our argument with current trends in EU governance reform.

Finally, we explicitly note that risk calibration should not only reflect external market volatility but also respond to individual-level motivational profiles. This adds a psychological dimension to governance policy that can be incorporated into board-level decision-making frameworks.

These enhancements clarify how boards and policymakers might design context-sensitive compensation architectures based on the interaction between institutional environment and executive psychology (see *Conclusions*, **pp. 61–62, lines 1320–1335**).

**Reviewer #1:** *(4.4.) The study does not explore boundary conditions, for example, how different governance regimes (e.g., shareholder vs. stakeholder-oriented firms) might influence the impact of compensation structures on CEO risk preferences.*

**Response:**
In the updated text, we explicitly call for future studies to investigate boundary conditions such as ownership models and national governance regimes. We discuss how stakeholder-oriented firms (e.g., in coordinated market economies) may respond differently to compensation structures than shareholder-driven firms. We also consider Poland’s hybrid system and suggest that governance salience may vary across institutional contexts, potentially moderating the motivational pathways we observed (see **p. 56, lines 1194–1203** of *Limitations and future research directions*).

**Reviewer #1:** *(5.) Narrow Geographic and Contextual Scope. (5.1.) The study claims to contribute to European corporate governance but focuses predominantly on Poland, without a comparative analysis of governance structures across different European economies.*

**Reviewer #2:** *(6.) The study focuses on Polish firms, but I wonder whether the findings can be generalized to other European corporate governance models (perhaps for authors’ future research). Are there structural differences that could influence CEO behavior in different markets? It would be interesting to see a continuation of this study on cross-country comparisons or industry-specific variations.*

**Response:**
While our empirical analysis is rooted in the Polish corporate context, we have revised the manuscript to more clearly position Poland as part of the broader Central and Eastern European (CEE) region, which is undergoing institutional convergence toward EU governance standards.

We now explicitly discuss how Poland’s hybrid governance model shares features with both Anglo-Saxon and stakeholder-oriented systems, making it a useful reference point for countries experiencing similar transformations. Furthermore, we highlight the limitations of generalization and encourage future cross-national studies to extend our framework to other European contexts with differing institutional logics (**p. 6, lines 137–145**).

**Reviewer #1:** *(5.2.) CEO compensation practices in Poland are compared to the US, but there is no discussion of intermediate models, such as the German dual-board system or Scandinavian stakeholder governance.*

**Response:**
We appreciate this observation and have expanded the discussion of European corporate governance models to include key distinctions between the Polish system and those found in Germany and Scandinavia. Specifically, we now describe how the German dual-board structure emphasizes co-determination and stakeholder representation, which may constrain risk-inducing incentives. Likewise, we highlight how Scandinavian governance prioritizes long-term value creation, low pay dispersion, and stewardship, particularly in family-controlled firms.

These institutional contrasts help contextualize our findings and clarify how different governance philosophies shape not only the structure of CEO compensation but also its behavioral interpretation by executives (**pp. 6–7, lines 146–158**). We believe this addition meaningfully addresses the reviewer’s request for a more comparative perspective.

**Reviewer #1:** *(5.3.) The role of institutional investors, activist shareholders, and governance reforms is underexplored, despite their influence on executive compensation policies.*

**Response:**
This issue has been addressed in the revised version of the *Discussion* section. We now examine the potential influence of institutional investors – particularly pension and insurance funds in Poland – as external moderators of CEO behavior. Drawing on examples from Germany and Scandinavia, we suggest that such actors may either constrain or reinforce the dispositional effects investigated in our study. We encourage further research into how ownership structure and shareholder activism influence incentive systems and shape CEOs’ risk-taking preferences (see **p. 56, lines 1204–1213**, section *Limitations and future research directions*).

**Reviewer #1:** *(6.) Lack of Practical and Policy Relevance. (6.1.) The study does not engage with real-world cases or industry examples to illustrate how compensation structures have affected CEO decision-making in practice.*

**Response:**
We fully agree that linking theory to practice enhances the contribution of empirical research. Accordingly, the revised *Discussion* section now integrates real-world examples to support the external validity of our findings. We draw parallels with the 2008 global financial crisis, during which uncapped short-term bonuses contributed to excessive risk-taking among promotion-focused executives in major financial institutions (Bannier & Feess, 2011). We also reference recent cases from the tech sector, where promotion-focused founder-CEOs with substantial equity holdings have undertaken high-risk, high-reward innovation projects. These examples are consistent with the interaction effects observed in our data and reinforce the theoretical model with empirical relevance (see **p. 51, lines 1090–1096**, *Discussion* section).

**Reviewer #1:** *(6.2.) The corporate governance recommendations are generic, failing to provide actionable insights for policymakers, investors, or corporate boards.*

**Response:**
This critique is well taken. In response, we have significantly strengthened the practical recommendations in the Discussion section. We now offer differentiated guidance based on CEOs’ motivational profiles. For instance, we recommend that promotion-focused CEOs be subject to compensation safeguards – such as capped bonuses, clawbacks, and deferred pay – to mitigate excessive risk-taking. Conversely, prevention-focused CEOs may benefit from moderate performance-based incentives to counterbalance their natural risk aversion.

We also propose board-level balancing strategies (e.g., pairing risk-prone CEOs with more conservative CFOs or board directors) and advocate for the integration of behavioral profiling into executive selection processes. These recommendations are now more specific, actionable, and closely aligned with the behavioral dynamics identified in our study (see **p. 52, lines 1102–1108**).

**Reviewer #1:** *(6.3.) There is no discussion of regulatory implications, for instance, how corporate governance reforms in the EU (e.g., shareholder rights directives) might shape CEO pay structures and risk-taking behavior.*

**Response:**
In the revised *Discussion* section, we now explicitly link our findings to ongoing regulatory developments within the European Union. In particular, we reference the Shareholder Rights Directive II (Directive (EU) 2017/828), which promotes transparency, shareholder engagement, and a long-term orientation in executive remuneration.

Our results support this policy direction by emphasizing the importance of designing compensation structures that align with CEOs’ psychological traits. We further propose incorporating behavioral governance training into board education programs to ensure that regulatory frameworks are grounded in empirical insights into how incentives interact with dispositional tendencies. This addition addresses both the regulatory context and its practical implications for governance reform (see **pp. 51–52, lines 1098–1102** of the *Discussion* section,).

**Reviewer #1:** *(7.) Quality of Communication and Structure. (7.1.) There is repetition in literature review and discussion sections, leading to redundancy.*

**Response:**
We thank the reviewer for this comment. In response, we have carefully revised the *Discussion* section to eliminate redundant theoretical definitions that were already presented in the literature review. Specifically, we removed the introductory summaries that repeated the conceptual foundations of regulatory focus theory (RFT), upper echelons theory (UET), and prospect theory (PT).

Instead, we now focus on interpreting the empirical findings through these theoretical lenses without reiterating their core assumptions (see **p. 50, lines 1054–1056** in the Discussion section,). As a result, the *Discussion* section remains focused on analytical interpretation and avoids unnecessary repetition of theoretical exposition.

**Reviewer #1:** *(7.2.) Tables and figures are not effectively integrated into the discussion, many results are simply restated rather than critically analyzed.*

**Response:**
In response, we have thoroughly revised the *Results* section, to better integrate Tables 1–3 and Supplementary Figures S2–S5 directly into the narrative, avoiding mere repetition of statistical coefficients. Instead, we now interpret the meaning, direction, and theoretical implications of the key coefficients within their empirical context.

For example, when discussing Model 3, we refer to Supplementary Figure S2 not only to illustrate the significant interaction effect between promotion focus and bonuses, but also to explore the underlying behavioral mechanisms (e.g., regulatory fit, overconfidence) that may account for the observed amplification effect. A similar interpretive integration has been applied to Supplementary Figures S3–S5.

We also discuss the within- and between-group variance explained (R² statistics), providing a more nuanced interpretation of the explanatory power of our models in light of prior behavioral strategy research. These improvements aim to enhance the coherence and analytical depth of the results narrative (see revised *Results* section, **pp. 32–47, lines 766–998**).

In addition, we have revised the Discussion section, substantially expanding and deepening our consideration of the implications of the findings (see *Discussion,* **pp. 47–52, lines 1001–1115**).
