# Supplementary material for: Promote or prevent? A regulatory focus perspective on managerial risk taking
Source: PLoS One. 2026 Jul 31;21(7):e0352905. doi: 10.1371/journal.pone.0352905 (PMC13426988; doi:10.1371/journal.pone.0352905)
Supplement: Attachment — Submitted filename: review.docx [file pone.0352905.s010.docx]

**Critical Review of the Article: "Heterogeneity in CEOs' Risk Attitudes and the Impact of Fixed and Variable Compensation Components in Corporate Governance Policies"**

This article explores the intersection of CEOs' psychological risk preferences and corporate governance mechanisms, specifically examining how fixed and variable compensation influence strategic risk-taking behaviors. The study employs **Regulatory Focus Theory (RFT)** and **Behavioral Agency Model (BAM)**, applying **longitudinal panel data** from **89 Polish companies (2011–2020)**. While the study presents an interesting approach, several critical weaknesses need to be addressed to ensure its suitability for publication.

**Lack of Clear Research Gap and Theoretical Contribution**

While the study discusses the role of regulatory focus in managerial decision-making, it does not sufficiently articulate its novelty relative to prior literature. The connection between CEO compensation and risk preferences has been widely studied within agency theory and behavioral finance, yet the paper does not explicitly delineate how its findings extend or challenge existing paradigms.

- The introduction lacks a critical synthesis of past studies and fails to clearly differentiate this study from prior empirical investigations.
- Although regulatory focus theory (RFT) is well-established in psychology and management research, the article does not integrate it with competing governance perspectives, such as the Upper Echelons Theory (UET) or Prospect Theory, which also address CEO decision-making biases. For instance, Sewpersadh (2019) examines different types of CEO power (structural, expert, prestige) and how these influence strategic decision-making. This provides a critical lens for evaluating **how CEO compensation aligns with different power structures**.

Sewpersadh, N.S. (2019), "An examination of CEO power with board vigilance as a catalyst for firm growth in South Africa", Measuring Business Excellence, Vol. 23 No. 4, pp. 377-395. https://doi.org/10.1108/MBE-10-2018-0083

- The study does not explore counterarguments or limitations of applying RFT in the CEO compensation context, leaving theoretical gaps unaddressed.

**2. Methodological Concerns**

The empirical design, while employing longitudinal panel data, has several shortcomings that undermine the robustness and generalizability of the findings.

**2.1 Sample and External Validity Issues**

- The study relies on a relatively small sample of 89 firms, predominantly from the Warsaw Stock Exchange (WSE), limiting its applicability to broader corporate governance contexts.
- The inclusion of a few firms from other European exchanges (e.g., Germany, Spain, Portugal) is inconsistent and lacks a clear justification.
- There is no discussion of industry-specific risk factors that might influence CEO decision-making differently across sectors.
- The omission of small and medium-sized enterprises (SMEs) creates a selection bias, as SMEs often face different governance and risk dynamics compared to publicly traded firms.

**2.2 Measurement Limitations**

- CEO regulatory focus is inferred through **content analysis of shareholder letters** using the Polish LIWC dictionary. However, the study does not critically engage with potential biases in this methodology.
  - The assumption that shareholder letters authentically reflect CEO decision-making is problematic, as such letters may be influenced by public relations teams or investor relations advisors.
  - The linguistic sentiment analysis approach may fail to capture deeper, contextual nuances in CEO motivation, leading to measurement error.
- Compensation structures are treated simplistically:
  - **Annual bonuses and fixed salaries** are analyzed, but the study does not account for long-term incentives, stock options, or equity-based compensation, which are common in executive pay structures.
  - The classification of **strategic risk-taking** using R&D expenditures, long-term debt, and CAPEX is overly simplistic, as it does not distinguish between calculated strategic investments and reckless risk-taking.
  - There is no sensitivity analysis to test whether alternative measures of risk-taking (e.g., acquisitions, internationalization strategies) would yield consistent results.

**2.3 Statistical Concerns**

- The study uses **fixed-effects panel regression models**, but there is no discussion of alternative specifications (e.g., random effects, instrumental variable approaches) to address potential endogeneity.
- The potential for **reverse causality** is not adequately controlled. It is plausible that firms with higher risk-taking tendencies select CEOs with a particular regulatory focus, rather than CEO traits solely driving firm behavior.
- Multicollinearity is cited as a reason for excluding control variables (CEO gender, tenure), but no robustness checks are provided to assess how their exclusion affects results.
- The Heckman two-step correction for sample selection bias is mentioned but not fully detailed, leaving uncertainty about its effectiveness in mitigating bias.

**3. Weaknesses in Hypothesis Development**

- The six hypotheses are **overly deterministic**, assuming linear relationships between regulatory focus, compensation, and risk-taking.
- The study **does not account for potential threshold effects**, for instance, at what level of compensation does risk-taking behavior significantly change?
- The moderating effects of fixed and variable compensation are discussed, but **no alternative models** (e.g., interaction effects with firm size or governance structures) are considered.
- Hypotheses are framed in **unidirectional terms**, without addressing conditions where relationships may not hold (e.g., economic downturns, regulatory changes).

**4. Superficial Discussion of Findings**

- The discussion section restates statistical findings but lacks critical engagement with **why certain hypotheses were not supported**.
- Unexpected findings, such as bonuses amplifying promotion-focused risk-taking rather than tempering it, are only briefly rationalized without deeper theoretical exploration.
- The **implications for policymakers and corporate boards** remain vague, with general recommendations rather than specific strategies for structuring CEO compensation in different governance environments.
- The study **does not explore boundary conditions**, for example, how different governance regimes (e.g., shareholder vs. stakeholder-oriented firms) might influence the impact of compensation structures on CEO risk preferences.

**5. Narrow Geographic and Contextual Scope**

- The study claims to contribute to European corporate governance but focuses predominantly on Poland, without a comparative analysis of governance structures across different European economies.
- CEO compensation practices in Poland are compared to the US, but **there is no discussion of intermediate models**, such as the German dual-board system or Scandinavian stakeholder governance.
- **The role of institutional investors, activist shareholders, and governance reforms** is underexplored, despite their influence on executive compensation policies.

**6. Lack of Practical and Policy Relevance**

- The study does not engage with real-world cases or industry examples to illustrate how compensation structures have affected CEO decision-making in practice.
- The corporate governance recommendations are **generic**, failing to provide actionable insights for policymakers, investors, or corporate boards.
- There is **no discussion of regulatory implications**, for instance, how corporate governance reforms in the EU (e.g., shareholder rights directives) might shape CEO pay structures and risk-taking behavior.

**7. Quality of Communication and Structure**

- There is **repetition in literature review and discussion sections**, leading to redundancy.
- Tables and figures are not **effectively integrated** into the discussion, many results are simply restated rather than critically analyzed.

These weaknesses reduce the study’s ability to make a meaningful contribution to corporate governance and strategic management literature.
