# Supplementary material for: Promote or prevent? A regulatory focus perspective on managerial risk taking
Source: PLoS One. 2026 Jul 31;21(7):e0352905. doi: 10.1371/journal.pone.0352905 (PMC13426988; doi:10.1371/journal.pone.0352905)
Supplement: Peer review history

# Peer review: Decision Letter 0

**DOI:** 10.1371/journal.pone.0352905.r001

**Authors:** Guy Hochman

4 Mar 2025

PONE-D-24-56730

Promote or prevent? A regulatory focus perspective on managerial risk taking

PLOS ONE

Dear Dr. Miązek,

Thank you for submitting your manuscript to PLOS ONE. After careful consideration, we feel that it has merit but does not fully meet PLOS ONE’s publication criteria as it currently stands. Therefore, we invite you to submit a revised version of the manuscript that addresses the points raised during the review process.

While Reviewer 2 is less critical, Reviewer 1 raises major concerns that must be addressed in the revised version. Both reviewers indicate the lack of clear goal and theoretical framework. However, I believe your data had mertis, and might be suitable for publications after you make major revisions.

Please submit your revised manuscript by Apr 18 2025 11:59PM. If you will need more time than this to complete your revisions, please reply to this message or contact the journal office at plosone@plos.org. When you're ready to submit your revision, log on to https://www.editorialmanager.com/pone/ and select the 'Submissions Needing Revision' folder to locate your manuscript file.

Please include the following items when submitting your revised manuscript:

- A rebuttal letter that responds to each point raised by the academic editor and reviewer(s). You should upload this letter as a separate file labeled 'Response to Reviewers'.
- A marked-up copy of your manuscript that highlights changes made to the original version. You should upload this as a separate file labeled 'Revised Manuscript with Track Changes'.
- An unmarked version of your revised paper without tracked changes. You should upload this as a separate file labeled 'Manuscript'.

If you would like to make changes to your financial disclosure, please include your updated statement in your cover letter. Guidelines for resubmitting your figure files are available below the reviewer comments at the end of this letter.

If applicable, we recommend that you deposit your laboratory protocols in protocols.io to enhance the reproducibility of your results. Protocols.io assigns your protocol its own identifier (DOI) so that it can be cited independently in the future. For instructions see: https://journals.plos.org/plosone/s/submission-guidelines#loc-laboratory-protocols. Additionally, PLOS ONE offers an option for publishing peer-reviewed Lab Protocol articles, which describe protocols hosted on protocols.io. Read more information on sharing protocols at https://plos.org/protocols?utm_medium=editorial-email&utm_source=authorletters&utm_campaign=protocols.

We look forward to receiving your revised manuscript.

Kind regards,

Guy Hochman, Ph.D.

Academic Editor

PLOS ONE

Journal requirements:

When submitting your revision, we need you to address these additional requirements.

1. Please ensure that your manuscript meets PLOS ONE's style requirements, including those for file naming. The PLOS ONE style templates can be found at

https://journals.plos.org/plosone/s/file?id=wjVg/PLOSOne_formatting_sample_main_body.pdf and

https://journals.plos.org/plosone/s/file?id=ba62/PLOSOne_formatting_sample_title_authors_affiliations.pdf

2. We notice that your supplementary [figures/tables] are included in the manuscript file. Please remove them and upload them with the file type 'Supporting Information'. Please ensure that each Supporting Information file has a legend listed in the manuscript after the references list.

Additional Editor Comments (if provided):

[Note: HTML markup is below. Please do not edit.]

Reviewers' comments:

Reviewer's Responses to Questions

**Comments to the Author**

1. Is the manuscript technically sound, and do the data support the conclusions?

The manuscript must describe a technically sound piece of scientific research with data that supports the conclusions. Experiments must have been conducted rigorously, with appropriate controls, replication, and sample sizes. The conclusions must be drawn appropriately based on the data presented.

Reviewer #1: No

Reviewer #2: Yes

**********

2. Has the statistical analysis been performed appropriately and rigorously?

Reviewer #1: No

Reviewer #2: Yes

**********

3. Have the authors made all data underlying the findings in their manuscript fully available?

The PLOS Data policy requires authors to make all data underlying the findings described in their manuscript fully available without restriction, with rare exception (please refer to the Data Availability Statement in the manuscript PDF file). The data should be provided as part of the manuscript or its supporting information, or deposited to a public repository. For example, in addition to summary statistics, the data points behind means, medians and variance measures should be available. If there are restrictions on publicly sharing data—e.g. participant privacy or use of data from a third party—those must be specified.

Reviewer #1: No

Reviewer #2: Yes

**********

4. Is the manuscript presented in an intelligible fashion and written in standard English?

PLOS ONE does not copyedit accepted manuscripts, so the language in submitted articles must be clear, correct, and unambiguous. Any typographical or grammatical errors should be corrected at revision, so please note any specific errors here.

Reviewer #1: Yes

Reviewer #2: Yes

**********

5. Review Comments to the Author

Please use the space provided to explain your answers to the questions above. You may also include additional comments for the author, including concerns about dual publication, research ethics, or publication ethics. (Please upload your review as an attachment if it exceeds 20,000 characters)

Reviewer #1: Well done on completing your research!

Your study provides a theoretically grounded investigation into the relationship between CEO regulatory focus, compensation, and strategic risk-taking. However, it falls short in several key areas:

Limited Theoretical Contribution – The study does not sufficiently advance existing research on CEO risk-taking and executive compensation.

Methodological Weaknesses – Sample limitations, measurement issues, and statistical concerns undermine the robustness of findings.

Over-Simplified Hypothesis Framing – The study assumes linear relationships and does not explore alternative explanations.

Superficial Discussion of Findings – The discussion fails to critically engage with contradictory results or real-world applications.

Narrow Geographic Focus – The study claims European relevance but primarily examines Polish firms without comparative insights.

Lack of Practical and Policy Impact – The study does not provide concrete recommendations for firms, regulators, or investors.

Communication and Structural Issues – The writing is repetitive, dense, and does not effectively integrate findings with theory.

My review report is attached that further explains each of the above areas for improvement.

Reviewer #2: It is a long manuscript but interesting to read, seems well-structured and offers some insights into Polish CEO decision-making and corporate governance.

Thank you for the opportunity to review this manuscript and please allow me to make some suggestions for the authors:

1. I believe that the theoretical foundation could better explain how and why different compensation structures impact risk-taking, perhaps with a clearer link between Regulatory Focus Theory and Behavioral Agency Theory

2. The use of shareholder letters to assess CEO regulatory focus is interesting, but I believe that some potential biases could be addressed. I wonder to what extent do these letters genuinely reflect CEO traits rather than corporate messaging strategies?

3. The authors measure strategic risk primarily through financial indicators (R&D spending, CAPEX, long-term debt), but I wonder whether including qualitative aspects like strategic expansion decisions could enhance the analysis? Perhaps in authors’ future studies they could include alternative measures for strategic risk, like market expansion, innovation strategies etc.

4. The finding that bonuses increase rather than temper risk-taking in promotion-focused CEOs challenges somehow the expectations. Is it possible the authors could give more details on alternative explanations, perhaps like intrinsic motivation or short-term financial goals?

5.Fixed salaries appear to reduce risk-taking in promotion-focused CEOs. I wonder if the authors could discuss a little bit governance or industry constraints that could explain this result.

6. The study focuses on Polish firms, but I wonder whether the findings can be generalized to other European corporate governance models (perhaps for authors’ future research). Are there structural differences that could influence CEO behavior in different markets? It would be interesting to see a continuation of this study on cross-country comparisons or industry-specific variations.

7. This is just a minor observation, about some statistical explanations in the results section are perhaps a little overly detailed and could be better placed in an appendix.

**********

6. PLOS authors have the option to publish the peer review history of their article (what does this mean?). If published, this will include your full peer review and any attached files.

If you choose “no”, your identity will remain anonymous but your review may still be made public.

**Do you want your identity to be public for this peer review?**  For information about this choice, including consent withdrawal, please see our Privacy Policy.

Reviewer #1: No

Reviewer #2: No

**********

[NOTE: If reviewer comments were submitted as an attachment file, they will be attached to this email and accessible via the submission site. Please log into your account, locate the manuscript record, and check for the action link "View Attachments". If this link does not appear, there are no attachment files.]

While revising your submission, please upload your figure files to the Preflight Analysis and Conversion Engine (PACE) digital diagnostic tool, https://pacev2.apexcovantage.com/. PACE helps ensure that figures meet PLOS requirements. To use PACE, you must first register as a user. Registration is free. Then, login and navigate to the UPLOAD tab, where you will find detailed instructions on how to use the tool. If you encounter any issues or have any questions when using PACE, please email PLOS at figures@plos.org. Please note that Supporting Information files do not need this step.

Attachment Submitted filename: review.docx

# Author comment: Author response to Decision Letter 1

**DOI:** 10.1371/journal.pone.0352905.r002

24 Jul 2025

Dear Reviewers,

We sincerely thank you for your detailed and constructive feedback. We have carefully addressed each comment and substantially revised the manuscript in line with the concerns raised. These revisions have resulted in significant improvements in the theoretical positioning, methodological transparency, and interpretive depth of our study. Below, we respond point-by-point to each comment. For each issue raised, we summarize our revisions and indicate where in the manuscript the relevant changes have been made (with page and line references, where applicable). We trust that these revisions adequately address your concerns and improve the quality and coherence of the manuscript.

Reviewer #1: (1.) Lack of Clear Research Gap and Theoretical Contribution. (1.1.) While the study discusses the role of regulatory focus in managerial decision-making, it does not sufficiently articulate its novelty relative to prior literature. The connection between CEO compensation and risk preferences has been widely studied within agency theory and behavioral finance, yet the paper does not explicitly delineate how its findings extend or challenge existing paradigms.

Reviewer #2: (1.) I believe that the theoretical foundation could better explain how and why different compensation structures impact risk-taking, perhaps with a clearer link between Regulatory Focus Theory and Behavioral Agency Theory.

Response:

We thank both reviewers for highlighting the need to better articulate the contribution of our study. We have revised the manuscript to emphasize the novelty of integrating RFT – a psychological framework – into the corporate governance literature, particularly in connection with compensation design. While previous studies (e.g., Sanders & Hambrick, 2007; Beatty & Zajac, 1994) examined the link between executive pay and risk-taking, few have explored how dispositional motivation moderates this relationship.

We now make clear that our study addresses this gap by demonstrating how RFT can explain behavioral heterogeneity in CEO responses to identical incentive structures. This approach advances existing theories (AT, BAM, UET) by accounting for individual-level variation in motivational orientation, a dimension often overlooked in governance research (pp. 5-6, lines 110–136).

Reviewer #1: (1.2.) The introduction lacks a critical synthesis of past studies and fails to clearly differentiate this study from prior empirical investigations.

Response:

We thank the reviewer for this insightful observation. In response, we have revised the Introduction section to provide a more explicit and critical synthesis of prior literature and to highlight how our study extends existing research in meaningful ways.

Specifically, we added a new integrative paragraph (see p. 20, lines 462–468) that outlines the empirical limitations of previous studies focusing on CEO risk-taking. We emphasize that prior research has largely relied on U.S.-centric data and focused either on structural variables (e.g., incentive alignment, power asymmetries) or dispositional traits in isolation, without systematically integrating motivational orientation (as conceptualized by Regulatory Focus Theory) with corporate governance mechanisms. Furthermore, we note that even when RFT is included in earlier studies (e.g., Gamache et al., 2015; Lanaj et al., 2012), it is typically treated as a secondary moderator rather than a core explanatory variable.

By embedding RFT within a broader theoretical framework that includes BAM, PT, and UET, and by testing this integration empirically in a hybrid governance environment (Poland), our study provides a cross-contextual, psychologically informed explanation of CEO strategic risk-taking. This approach goes beyond prior research by demonstrating how the same formal incentives may elicit divergent behaviors depending on stable motivational orientation. Thus, we now clearly position our contribution as a theoretically novel and empirically grounded advancement in the behavioral governance literature.

We hope these revisions directly address the reviewer’s concern and clarify how our study builds upon and differentiates itself from earlier empirical investigations.

Reviewer #1: (1.3.) Although regulatory focus theory (RFT) is well-established in psychology and management research, the article does not integrate it with competing governance perspectives, such as the Upper Echelons Theory (UET) or Prospect Theory, which also address CEO decision-making biases. For instance, Sewpersadh (2019) examines different types of CEO power (structural, expert, prestige) and how these influence strategic decision-making. This provides a critical lens for evaluating how CEO compensation aligns with different power structures. Sewpersadh, N.S. (2019), "An examination of CEO power with board vigilance as a catalyst for firm growth in South Africa", Measuring Business Excellence, Vol. 23 No. 4, pp. 377-395. https://doi.org/10.1108/MBE-10-2018-0083

Response:

We appreciate the reviewer’s suggestion to more clearly situate Regulatory Focus Theory (RFT) within the broader landscape of executive decision-making frameworks. In response, we have revised the theoretical section to integrate RFT with both Upper Echelons Theory (UET) and Prospect Theory (PT). Specifically, we now clarify that RFT captures stable, chronic motivational orientations (promotion vs. prevention focus), whereas UET emphasizes observable executive traits and PT focuses on situational framing of decisions. By aligning RFT with these established frameworks, we offer a complementary psychological lens that enriches our understanding of CEO-level variability in risk preferences under identical structural conditions (pp. 3–4, lines 63–88).

Additionally, we explicitly contrast our dispositional approach with power-based governance models such as Sewpersadh (2019), emphasizing that our contribution shifts the analytical focus from positional authority to internal motivational states. We believe this integration addresses the reviewer’s concern and strengthens the theoretical coherence of the study.

Reviewer #1: (1.4.) The study does not explore counterarguments or limitations of applying RFT in the CEO compensation context, leaving theoretical gaps unaddressed.

Response:

In the revised Discussion, we explicitly address potential limitations of applying RFT to CEO behavior. We note that while RFT offers a robust dispositional lens, it does not account for all situational constraints imposed by organizational governance or external monitoring. For instance, the expected moderating effects of compensation on promotion-focused CEOs were not supported, suggesting that financial incentives may be less influential in shaping risk behavior for this group. We now interpret this as a possible boundary condition for the applicability of RFT in high-status executive roles, where agency discretion may be tempered by power dynamics or pre-existing strategic mandates (see pp. 49–50, lines 1044–1053 in Discussion). We have also signaled the need for future research to test whether RFT effects hold under varying institutional logics and leadership contexts.

Reviewer #1: (2.) Methodological Concerns. The empirical design, while employing longitudinal panel data, has several shortcomings that undermine the robustness and generalizability of the findings. (2.1) Sampling and external validity. (2.1.1.) The study relies on a relatively small sample of 89 firms, predominantly from the Warsaw Stock Exchange (WSE), limiting its applicability to broader corporate governance contexts.

Response:

In the revised manuscript, we explicitly acknowledge this limitation (see p. 52, lines 1019–1020, section: Limitations and future research directions), clarifying that the final sample of 82 firms (resulting from the exclusion of 7 companies listed on non-Polish stock exchanges – see reviewer comment below) reflects a data-driven sampling logic (see also p. 21, lines 497–506, and p. 22, lines 509–511). This logic prioritizes the availability of complete and high-quality data (CEO letters, compensation, and strategic indicators) over full population coverage. Such a sampling approach is common in management and behavioral research, where disclosure practices vary significantly across jurisdictions.

In Poland, there is no strict obligation to disclose many types of corporate information beyond core financial statements for publicly listed companies. However, recent legal and market developments indicate a clear trend towards greater transparency. Limited access to data that is widely available in other jurisdictions, such as the United States, poses a serious challenge for researchers in many countries, including those in Central and Eastern Europe. This affects the size of usable, high-quality samples and represents both a limitation and a structural research challenge for scholars working in these contexts.

To mitigate potential sample selection bias arising from non-random availability of CEO letters and compensation data – which may be correlated with unobserved determinants of strategic risk – we applied the two-step Heckman correction procedure (Heckman, 1979). This procedure, described in detail later in our response, allowed us to generate the inverse Mills ratio (IMR), which was included in both the fixed effects (FE) and random effects (RE) models (see Method section).

Although the primary empirical focus is on Poland, the findings can be analytically extended to other continental European governance systems that share similar institutional characteristics and a hybrid corporate model (p. 56, lines 1194–1203, section: Limitations and future research directions). The study contributes to the growing body of research on executive behavior outside the United States, particularly within the underrepresented context of Central and Eastern Europe.

Reviewer #1: (2.1.2.) The inclusion of a few firms from other European exchanges (e.g., Germany, Spain, Portugal) is inconsistent and lacks a clear justification.

Response:

In the revised version of the manuscript (see p. 21, lines 497–506, and p. 22, lines 509–511), we decided to exclude the 7 companies listed outside the Polish stock exchange. Their initial inclusion was motivated by the availability of comparable longitudinal data on CEO communication and compensation, given the significant data gaps observed among companies listed on the Warsaw Stock Exchange (WSE), particularly regarding remuneration, bonuses, and CEO letters to shareholders. However, upon further reflection, we fully agree with the reviewers that such a mixed sample lacks consistency and that the justification for its inclusion was insufficient.

Reviewer #1: (2.1.3.) There is no discussion of industry-specific risk factors that might influence CEO decision-making differently across sectors.

Reviewer #2: (5.) Fixed salaries appear to reduce risk-taking in promotion-focused CEOs. I wonder if the authors could discuss a little bit governance or industry constraints that could explain this result.

Response:

In the revised version of the manuscript, we have included a sector control variable and indicated the distribution of companies in the sample across 11 sectors (as detailed in the sample description on p. 22, lines 512–521 and in Supplementary Table S1). Unfortunately, this variable was automatically excluded from the main fixed effects (FE) models due to its time-invariant nature, which is a known limitation of FE estimation. However, it was included in the robustness check using a random effects (RE) model, where it did not display a statistically significant relationship with the dependent variable – managerial strategic risk.

This does not imply that sectoral context is irrelevant in our research. We fully acknowledge that limitations of the sample prevented a proper examination of sectoral conditioning, and our model does not fully capture sector-specific risk norms. We recognize this as a limitation and emphasize the need for more in-depth treatment of this variable in future studies. This limitation is illustrated by contrasting highly regulated sectors (e.g., banking) with less regulated ones (e.g., technology). We also highlight that sectoral conventions are likely to shape how CEOs perceive strategic boldness – either as innovation or recklessness – depending on the industry context. This insight provides a foundation for future research involving sector-specific sampling or interaction terms (see pp. 53–54, lines 1134–1148 in the section Limitations and future research directions).

Reviewer #1: (2.1.4.) The omission of small and medium-sized enterprises (SMEs) creates a selection bias, as SMEs often face different governance and risk dynamics compared to publicly traded firms.

Response:

We acknowledge this limitation and have explicitly addressed it in the revised manuscript. In the updated section, we now clarify that the sample includes only publicly listed firms and discuss how this introduces selection bias, as SMEs may exhibit distinct governance mechanisms and risk dynamics (Berger & Udell, 2006). We also highlight that CEO ownership is typically higher in SMEs, which may amplify or dampen the effects of regulatory focus. We encourage future studies to examine whether our findings replicate in SME contexts, where incentive structures are often less formalized and leadership effects more personalized (see p. 53, lines 1125–1133 of Limitations and future research directions).

Reviewer #1: (2.2) Measurement Limitations. CEO regulatory focus is inferred through content analysis of shareholder letters using the Polish LIWC dictionary. However, the study does not critically engage with potential biases in this methodology. (2.2.1.) The assumption that shareholder letters authentically reflect CEO decision-making is problematic, as such letters may be influenced by public relations teams or investor relations advisors.

Reviewer #2: (2.) The use of shareholder letters to assess CEO regulatory focus is interesting, but I believe that some potential biases could be addressed. I wonder to what extent do these letters genuinely reflect CEO traits rather than corporate messaging strategies?

Response:

We appreciate the reviewers’ concerns regarding the potential limitations of using shareholder letters to infer CEOs’ regulatory focus. In response, we have added a more extensive description of the content analysis methodology in the section detailing the measurement of our key psychological variables, including a justification for the choice of this method (p. 23, lines 538–545), and we explicitly address the possible influence of investor relations or public relations teams on the texts. We acknowledge that these publicly disclosed documents may not fully reflect unfiltered cognitive states. However, we clarify that our analysis aims to capture the externally communicated motivational emphasis of CEOs, rather than introspective content (see also the section Limitations and future research directions, p. 54, lines 1149–1162).

To strengthen the validity of our approach, we highlight that the Polish LIWC-based dictionary of regulatory orientation used in this study has been empirically validated in multiple studies, demonstrating content, discriminant, and correlational validity (Marszałek et al., 2023). This makes it a psychometrically robust tool for detecting promotion and prevention focus in Polish-language texts.

We also reinforce our methodological rationale by drawing on prior research (e.g., Gamache et al., 2015; Zheng et al., 2020), which successfully employed managerial discourse to infer dispositional traits. Moreover, we note that CEOs typically participate in drafting or thoroughly editing these letters and bear legal responsibility for their content, which supports the assumption that the language used reflects their strategic perspectives and motivational priorities.

In addition, we cite literature showing that linguistic style in corporate communication reflects stable psychological dispositions (Tausczik & Pennebaker, 2010), and that shareholder letters provide a discreet and consistent medium for long-term psychometric assessment (e.g., Craig & Amernic, 2011; Yekini et al., 2019

Attachment Submitted filename: Response to Reviewers.docx

# Peer review: Decision Letter 1

**DOI:** 10.1371/journal.pone.0352905.r003

**Authors:** Annesha Sil

17 Jun 2026

Promote or prevent? A regulatory focus perspective on managerial risk taking

PONE-D-24-56730R1

Dear Dr. Miązek,

We’re pleased to inform you that your manuscript has been judged scientifically suitable for publication and will be formally accepted for publication once it meets all outstanding technical requirements.

Within one week, you’ll receive an e-mail detailing the required amendments. When these have been addressed, you’ll receive a formal acceptance letter and your manuscript will be scheduled for publication.

An invoice will be generated when your article is formally accepted. Please note, if your institution has a publishing partnership with PLOS and your article meets the relevant criteria, all or part of your publication costs will be covered. Please make sure your user information is up-to-date by logging into Editorial Manager at Editorial Manager® and clicking the ‘Update My Information' link at the top of the page. For questions related to billing, please contact billing support.

If your institution or institutions have a press office, please notify them about your upcoming paper to help maximize its impact. If they’ll be preparing press materials, please inform our press team as soon as possible -- no later than 48 hours after receiving the formal acceptance. Your manuscript will remain under strict press embargo until 2 pm Eastern Time on the date of publication. For more information, please contact onepress@plos.org.

Kind regards,

Annesha Sil, Ph.D.

Staff Editor

PLOS One

Additional Editor Comments (optional):

Reviewers' comments:

Reviewer's Responses to Questions

**Comments to the Author**

1. If the authors have adequately addressed your comments raised in a previous round of review and you feel that this manuscript is now acceptable for publication, you may indicate that here to bypass the “Comments to the Author” section, enter your conflict of interest statement in the “Confidential to Editor” section, and submit your "Accept" recommendation.

Reviewer #2: All comments have been addressed

**********

2. Is the manuscript technically sound, and do the data support the conclusions?

The manuscript must describe a technically sound piece of scientific research with data that supports the conclusions. Experiments must have been conducted rigorously, with appropriate controls, replication, and sample sizes. The conclusions must be drawn appropriately based on the data presented.

Reviewer #2: Yes

**********

3. Has the statistical analysis been performed appropriately and rigorously?

Reviewer #2: Yes

**********

4. Have the authors made all data underlying the findings in their manuscript fully available?

The PLOS Data policy requires authors to make all data underlying the findings described in their manuscript fully available without restriction, with rare exception (please refer to the Data Availability Statement in the manuscript PDF file). The data should be provided as part of the manuscript or its supporting information, or deposited to a public repository. For example, in addition to summary statistics, the data points behind means, medians and variance measures should be available. If there are restrictions on publicly sharing data—e.g. participant privacy or use of data from a third party—those must be specified.

Reviewer #2: Yes

**********

5. Is the manuscript presented in an intelligible fashion and written in standard English?

PLOS ONE does not copyedit accepted manuscripts, so the language in submitted articles must be clear, correct, and unambiguous. Any typographical or grammatical errors should be corrected at revision, so please note any specific errors here.

Reviewer #2: Yes

**********

6. Review Comments to the Author

Please use the space provided to explain your answers to the questions above. You may also include additional comments for the author, including concerns about dual publication, research ethics, or publication ethics. (Please upload your review as an attachment if it exceeds 20,000 characters)

Reviewer #2: From what I see in the revised version of the manuscript, all my previous comments have been addressed in a constructive way. The theoretical foundation has been clarified (especially the link between Regulatory focus theory and Behavioral agency Theory) and the methodology has been strengthened. The authors have also acknowledged the limitations of using shareholder letters and financial indicators and they also added robustness checks where possible.

I appreciate the new discussions on motivational alignment, governance variation, the cross-national relevance of the findings. I consider that the revised paper is significantly stronger

**********

7. PLOS authors have the option to publish the peer review history of their article (what does this mean?). If published, this will include your full peer review and any attached files.

If you choose “no”, your identity will remain anonymous but your review may still be made public.

**Do you want your identity to be public for this peer review?**  For information about this choice, including consent withdrawal, please see our Privacy Policy.

Reviewer #2: No

**********

# Editor report: Acceptance letter

**DOI:** 10.1371/journal.pone.0352905.r004

**Authors:** Annesha Sil

PONE-D-24-56730R1

PLOS One

Dear Dr. Miązek,

I'm pleased to inform you that your manuscript has been deemed suitable for publication in PLOS One. Congratulations! Your manuscript is now being handed over to our production team.

At this stage, our production department will prepare your paper for publication. This includes ensuring the following:

* All references, tables, and figures are properly cited

* All relevant supporting information is included in the manuscript submission,

* There are no issues that prevent the paper from being properly typeset

You will receive further instructions from the production team, including instructions on how to review your proof when it is ready. Please keep in mind that we are working through a large volume of accepted articles, so please give us a few days to review your paper and let you know the next and final steps.

Lastly, if your institution or institutions have a press office, please let them know about your upcoming paper now to help maximize its impact. If they'll be preparing press materials, please inform our press team within the next 48 hours. Your manuscript will remain under strict press embargo until 2 pm Eastern Time on the date of publication. For more information, please contact onepress@plos.org.

You will receive an invoice from PLOS for your publication fee after your manuscript has reached the completed accept phase. If you receive an email requesting payment before acceptance or for any other service, this may be a phishing scheme. Learn how to identify phishing emails and protect your accounts at https://explore.plos.org/phishing.

If we can help with anything else, please email us at customercare@plos.org.

Thank you for submitting your work to PLOS ONE and supporting open access.

Kind regards,

PLOS ONE Editorial Office Staff

on behalf of

Dr Annesha Sil

Staff Editor

PLOS One
